# Supplementary material for: Alkylamine-tethered molecules recruit FBXO22 for targeted protein degradation
Source: Nat Commun. 2024 Jun 26;15:5409. doi: 10.1038/s41467-024-49739-3 (PMC11208438; doi:10.1038/s41467-024-49739-3)
Supplement: Supplementary file 1 — Supplementary Information [file 41467_2024_49739_MOESM1_ESM.pdf]

Supplementary Information

Alkylamine-tethered molecules recruit FBXO22 for targeted protein degradation

Authors

Chrysanthi Kagiou[1], Jose Antonio Cisneros[1], Jakob Farnung[2], Joanna Liwocha[2], Fabian Offensperger[1], Kevin Dong[3], Ka Yang[3], Gary Tin[1], Christina S. Horstmann[1,4], Matthias Hinterndorfer[1], Joao A. Paulo[3], Natalie S. Scholes[1], Juan Sanchez Avila[1], Michaela Fellner[5], Florian Andersch[5], J. Thomas Hannich[1], Johannes Zuber[5], Stefan Kubicek[1], Steven P. Gygi[3], Brenda A. Schulman[2], Georg E. Winter[1,#]

Affiliations

[1]CeMM Research Center for Molecular Medicine of the Austrian Academy of Sciences, 1090 Vienna, Austria

[2]Department of Molecular Machines and Signaling, Max Planck Institute of Biochemistry, Am Klopferspitz 18, 82152, Martinsried, Germany

[3]Department of Cell Biology, Harvard Medical School, Boston, MA, USA

[4]St. Anna Children's Cancer Research Institute, Vienna, Austria

[5]Research Institute of Molecular Pathology, Vienna BioCenter, 1030 Vienna, Austria.

[#]correspondence to gwinter@cemm.oeaw.ac.at

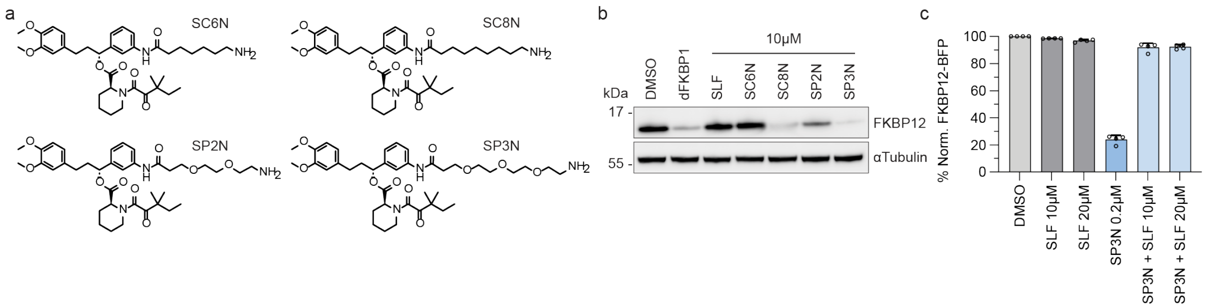

Supplementary Fig. 1 Alkylamine-tethered SLF induces FKBP12 degradation dependent on the primary amine.

a Structures of different alkylamines attached to SLF, SC6N: SLF-C6-NH2; SC8N: SLF-C8-NH2; SP2N: SLF-PEG2-NH2; SP3N: SLF-PEG3-NH2. **b** Immunoblot of FKBP12 in HEK293T-Nluc-3xFlag-FKBP12-NLS cells treated with DMSO, 1 µM dFKBP1, or 10 µM SLF, SC6N, SC8N, SP2N or SP3N. αTubulin is the loading control. Representative blot of n=3 independent experiments. **c** Flow-cytometry based degradation assay for SP3N + SLF competition. KBM7 iCas9 cells expressing the FKBP12-BFP-P2A-mCherry reporter were treated with DMSO, SLF (10 or 20 µM) or 200 nM SP3N individually or in combination, for 16 h. The BFP/mCherry ratio was normalized to DMSO. Mean ± s.d. of n=4 independent experiments.

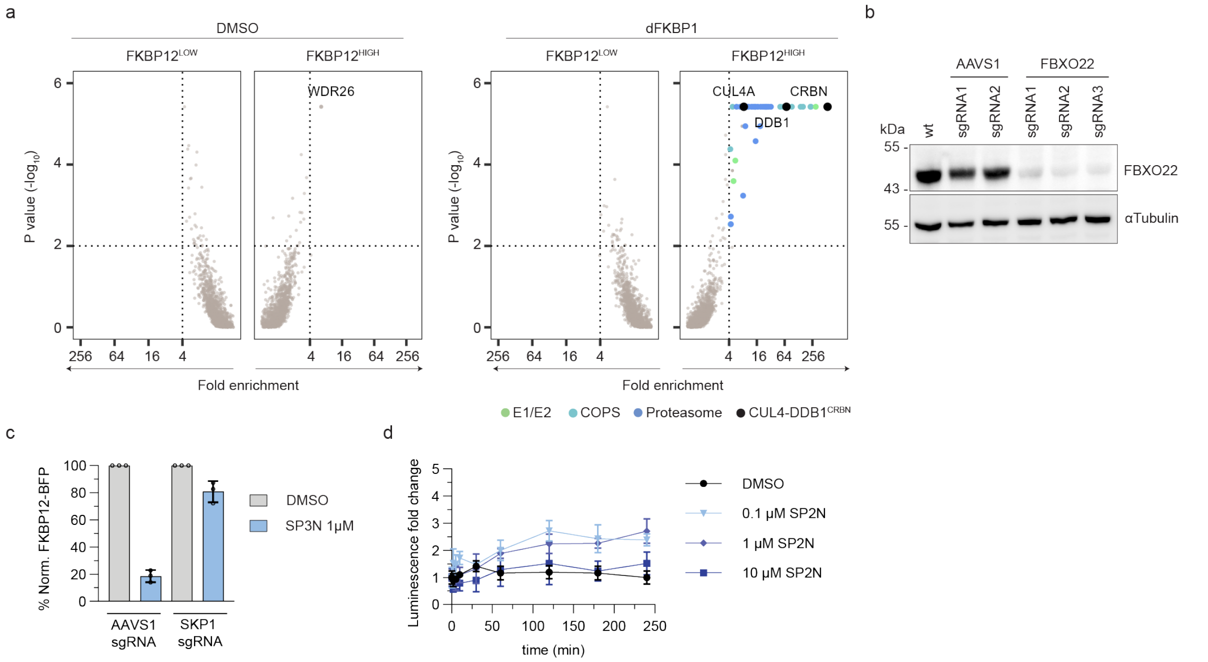

Supplementary Fig. 2 Alkylamine-tethered molecules recruit FBXO22 for FKBP12 degradation.

a FACS-based CRISPR/Cas9 stability screen as described in Fig. 2a for control treatments with DMSO or 100 nM dFKBP1 (16h). Hits with fold-enrichment ≥ 4 and -log$_{10}$Pvalues ≥ 2 (calculated using two-sided negative binomial test with MAGeCK) are highlighted as significant (dotted gray lines). Data from n=2 replicates. **b** Immunoblot for FBXO22 levels in KBM7 iCas9 FKBP12-BFP-P2A-mCherry reporter cells with AAVS1 or FBXO22 targeting sgRNAs. WT cells are without any sgRNA. αTubulin is the loading control. Representative blot of n=2 independent experiments. **c** Flow cytometry-based degradation assay for screen validation. KBM7 iCas9 FKBP12-BFP-P2A-mCherry reporter cells transduced with sgRNAs targeting either the control locus AAVS1 or SKP1 and treated with DMSO or 1 µM SP3N for 16 h. The BFP/mCherry ratio was normalized to DMSO. Mean ± s.d. of n=3 independent experiments **d** NanoBiT assay in HEK293T cells co-transfected with LgBiT-FKBP12 and SmBiT-FBXO22 and treated with DMSO or 0.1, 1 or 10 µM SP2N. The luminescence was monitored after 2 min, 5min, 10 min, 30 min and every hour up to 4 h post-treatment. Fold-change is calculated based on the luminescence at timepoint 0, right before the treatments. Mean ± s.d of n=3 technical replicates; representative of n=3 independent experiments.

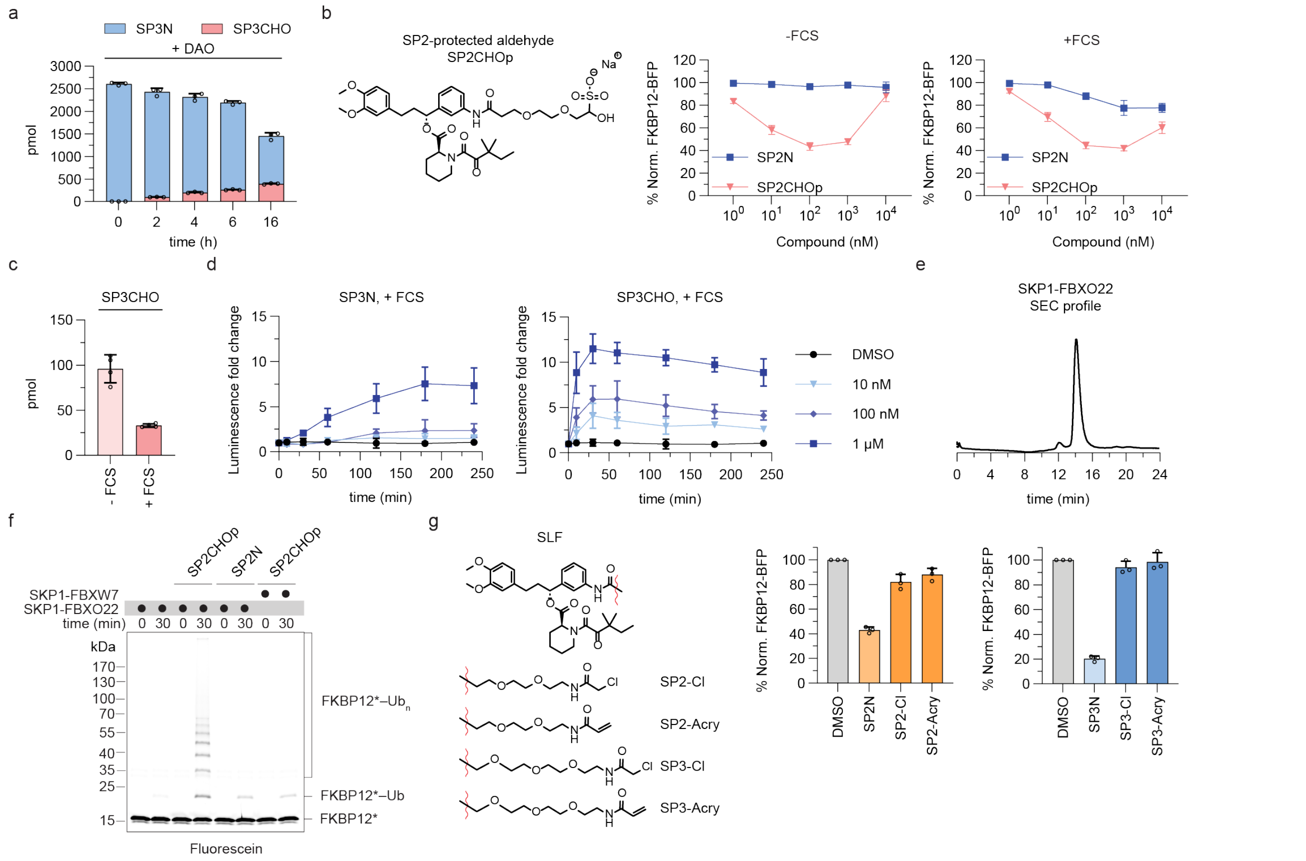

Supplementary Fig. 3 The alkylamines are metabolized to active aldehydes.
a Quantification of SP3N and SP3CHO (pmol) using UPLC-MS/MS, in PBS. 10 µM SP3N were added in PBS with 40 µg DAO and incubated for 0-16 h at 37 °C. Conditions without DAO were used as controls. Mean ± s.d. of n=3 technical replicates. **b** Flow-cytometry based degradation assay in KBM7 iCas9 with the FKBP12-BFP-P2A-mCherry reporter washed to remove FCS, resuspended in IMDM + 10% FCS or Opti-MEM - FCS and treated with DMSO, SP2N or SP2CHO protected aldehyde adduct (SP3CHOp) at the indicated concentrations for 6 h. **c** Quantification of intracellular SP3CHO (pmol) in medium -/+ FCS. KBM7 iCas9 FKBP12-BFP-P2A-mCherry FBXO22 KO cells were treated with 1 µM SP3CHO in OptiMEM -/+ FCS for 5 min and the SP3CHO levels were quantified using UPLC-MS/MS. Mean ± s.d. of n=4 technical replicates. **d** NanoBiT assay as described in Fig. 3f. Before treatment, the cells were washed 3x with PBS and the indicated concentrations of SP3N or SP3CHO were added to the cells in Opti-MEM + 10% FCS. The luminescence was monitored after 10 min, 30 min and every hour up to 4h post-treatment. Fold-change is calculated based on the luminescence at timepoint 0, right before the treatments. Mean ± s.d of n=3 technical replicates; representative of n=3 independent experiments. **e** Size exclusion chromatography (SEC) profile of the recombinant SKP1-FBXO22-WT. Relative absorbance over time is shown. **f** *In vitro* multi-turnover ubiquitylation assay of fluorescently labeled FKBP12 with SKP1-FBXO22 in the presence of DMSO, 10 µM SP2N or 10 µM SP2CHOp. SKP1-FBXW7 was used as negative control. Representative blot of n=2 independent experiments. **g** Flow-cytometry based degradation assay in KBM7 iCas9 with the FKBP12-BFP-P2A-mCherry reporter treated with DMSO or 10 µM SP2N, SP2-acrylamide (SP2-Acry), SP2-chloroacetamide (SP2-Cl), SP3N, SP3-Acry or SP3-Cl for 16 h. For all the flow-cytometry based degradation assays (b, g), the BFP/mCherry ratio was normalized to DMSO and the data is the mean ± s.d. from n = 3 biological replicates.

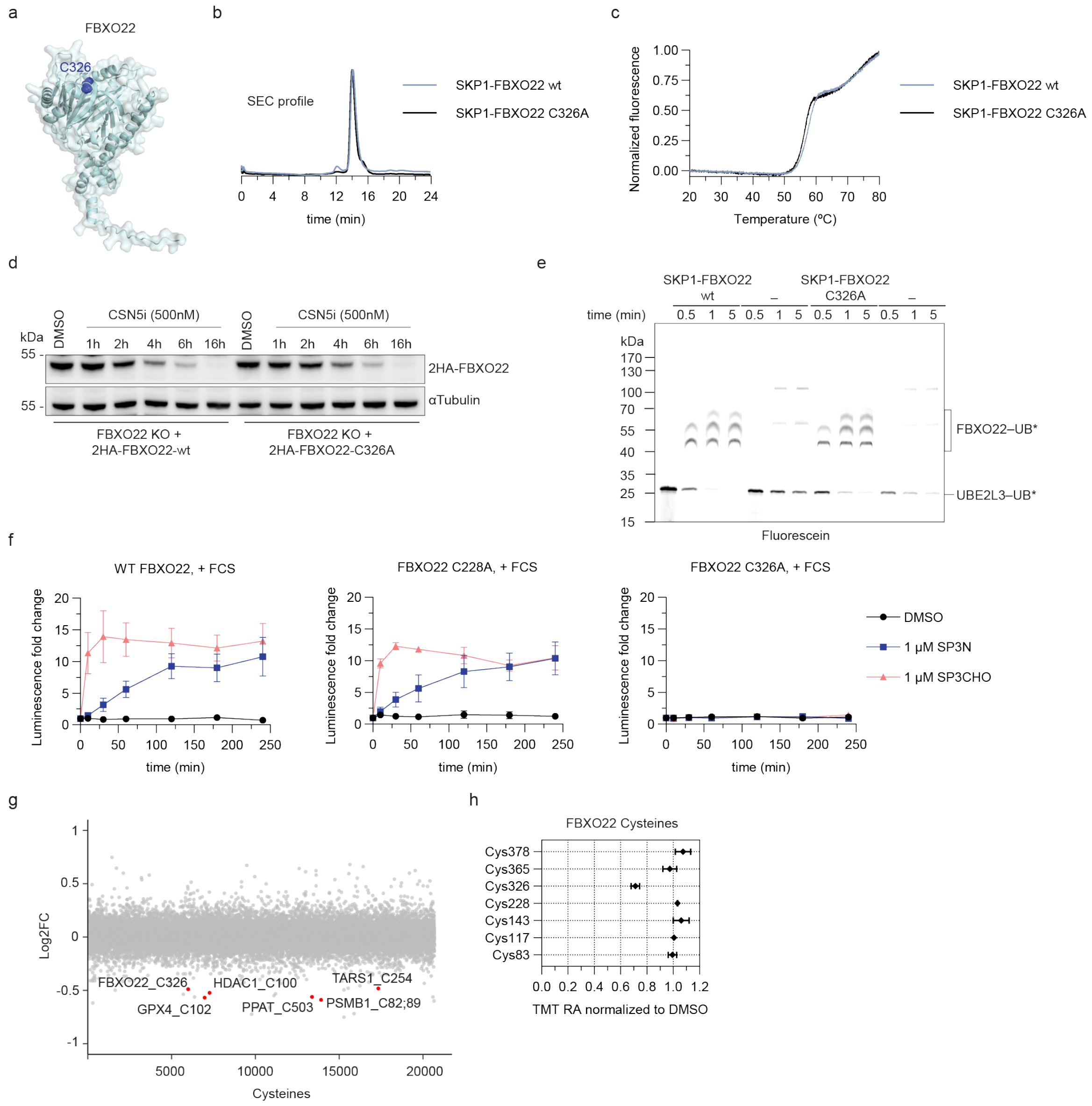

Supplementary Fig. 4 FBXO22-C326 is crucial for the degradation induced by SP3N/SP3CHO.
a AlphaFold prediction of FBXO22 structure with C326 highlighted in blue. **b** Comparison of SEC profiles of SKP1-FBXO22-WT and SKP1-FBXO22-C326A. Relative absorbance over time is shown. **c** Normalized fluorescence data from nano differential scanning fluorimetry (NanoDSF) of SKP1-FBXO22-WT and SKP1-FBXO22-C326A. Heat denaturation was measured at a rate of 1 °C/min. Measurements were performed in duplicates. **d** Immunoblot of 2HA-FBXO22-WT or 2HA-FBXO22-C326A in HEK293T-FKBP12-BFP-P2A-mCherry FBXO22 KO single clone transduced with 2HA-FBXO22-WT or -C326A cDNAs. Cells were treated with DMSO or 500 nM CSN5i-03 for 1-16 h. αTubulin is the loading control. Representative plot of n=2 independent experiments. **e** Pulse-chase *in vitro* autoubiquitylation assay of FBXO22 with SKP1-FBXO22-WT and SKP1-FBXO22-C326A. UB*: fluorescent ubiquitin. Representative blot from n=2 independent experiments. **f** NanoBiT assay in HEK293T cells co-transfected with LgBiT-FKBP12 and SmBiT-FBXO22-WT, SmBiT-FBXO22-C228A or SmBiT-FBXO22-C326A and treated with DMSO, 1 μM SP3N or 1 μM SP3CHO. The luminescence was monitored after 10 min, 30 min and every hour up to 4 h post-treatment. Mean ± s.d of n=3 technical replicates; representative of n=3 independent experiments. **g** Proteome-wide TMT-ABPP profiling of SP3CHO in HEK293T cell lysates spiked with 0.15 μg recombinant SKP1-FBXO22 and treated with 40 μM SP3CHO for 1.5 h. More than 20,000 cysteine sites were quantified. Log2 fold-changes (Log2FC) were calculated based on the DMSO treated cells. Red dots represent cysteine sites with Log2FC < -0.45 and -logPvalue > 2. Data from n=3 replicates. **h** TMT relative abundances (RA) of the quantified FBXO22 cysteines in the TMT-ABPP as described in g. RA are normalized to DMSO. Mean ± s.d. of n=3 replicates.

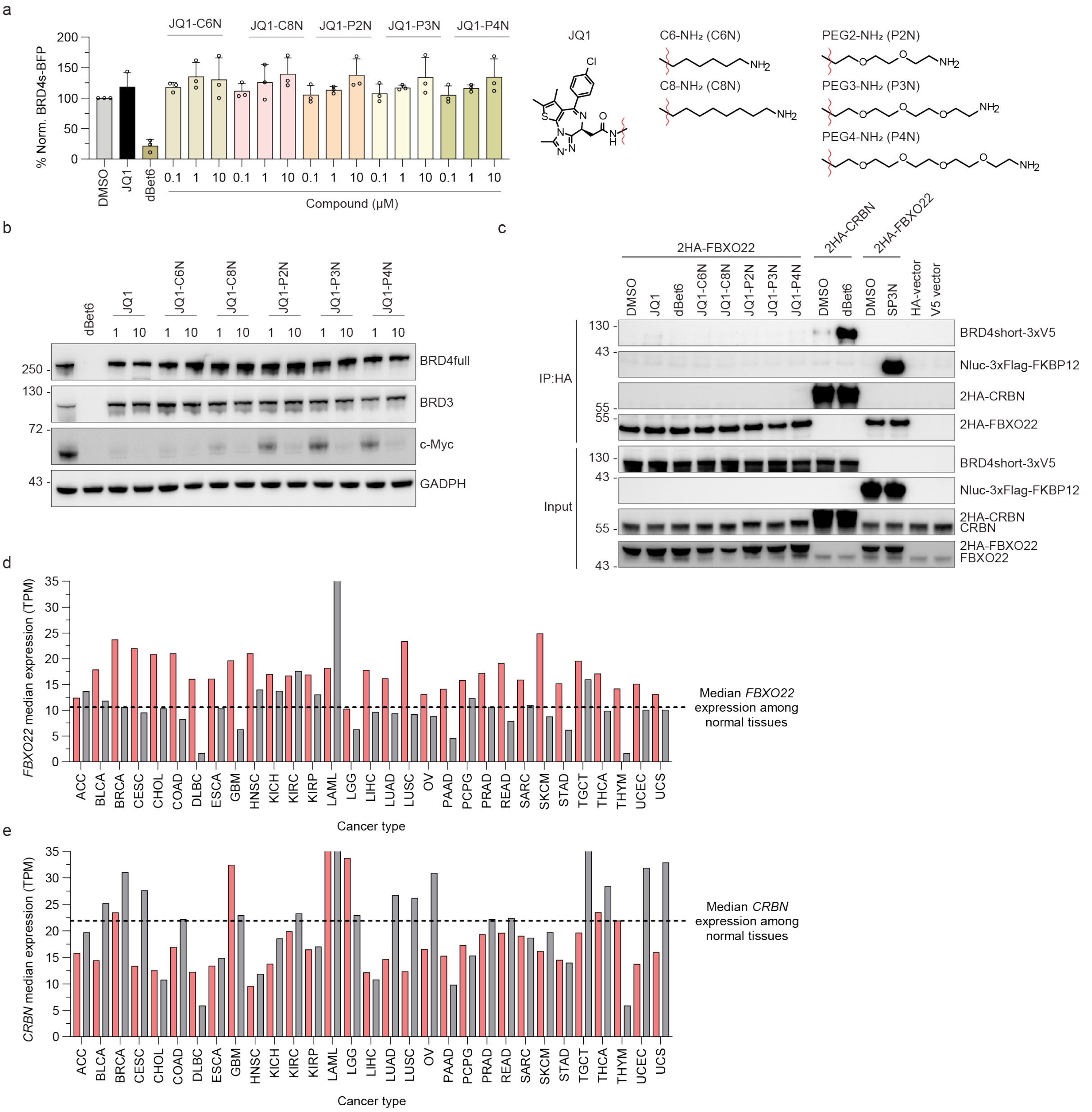

Supplementary Fig. 5 Expandability of co-opting FBXO22.
a Flow-cytometry based degradation assay in KBM7 iCas9 BRD4(short)-BFP-P2A-mCherry cells treated with DMSO, 10 µM JQ1, 1 µM dBet6 or the indicated concentrations of the different alkylamine-tethered JQ1 molecules for 16 h. Mean ± s.d. of n=3 biological replicates. **b** Immunoblot of endogenous BRD4, BDR3 and c-Myc in KBM7 iCas9 FKBP12-BFP-P2A-mCherry cells treated with DMSO, 1 µM dBet6, 1 or 10 µM JQ1 and 1 µM or 10 µM of the JQ1-alkylamines for 16 h. GADPH is the loading control. Representative blot of n=2 experiments. **c** Co-immunoprecipitation of 2HA-FBXO22 and BRD4s-3xV5 following treatment with DMSO, 1 µM dBet6, 10 µM JQ1 or 10 µM of the indicated JQ1-alkylamines, for 4 h in the presence of 1 µM carfilzomib. Co-immunoprecipitation of 2HA-FBXO22 with Nluc-3xFlag-FKBP12 upon treatment with 10 µM SP3N or of 2HA-CRBN with BRD4s-3xV5 upon treatment with 1 µM dBet6 were used as positive controls. Transfection with only HA-empty vector or V5-empty vector were used as negative controls for the IPs. Representative blot of n=2 experiments. IP: immunoprecipitation, IB: immunoblot. **d, e** Comparisons of the median gene expression (TPM; transcripts per million) of *FBXO22* (d) or *CRBN* (e) in different cancer tissues and the respective normal tissues extracted from TCGA and GTEx using GEPIA2. Gray bars: Normal tissues. Red bars: Cancer tissues. Dotted horizontal line: the median *FBXO22* or *CRBN* levels among all healthy tissues. ACC, Adrenocortical carcinoma; BLCA, Bladder Urothelial Carcinoma; BRCA, Breast invasive carcinoma; CESC, Cervical squamous cell carcinoma and endocervical adenocarcinoma; CHOL, Cholangio carcinoma; COAD, Colon adenocarcinoma; DLBC, Lymphoid Neoplasm Diffuse Large B-cell Lymphoma; ESCA, Esophageal carcinoma; GBM, Glioblastoma multiforme; HNSC, Head and Neck squamous cell carcinoma; KICH, Kidney Chromophobe; KIRC, Kidney renal clear cell carcinoma; KIRP, Kidney renal papillary cell carcinoma; LAML, Acute Myeloid Leukemia; LGG, Brain Lower Grade Glioma;

LIHC, Liver hepatocellular carcinoma; LUAD, Lung adenocarcinoma; LUSC, Lung squamous cell carcinoma; OV, Ovarian serous cystadenocarcinoma; PAAD Pancreatic adenocarcinoma; PCPG, Pheochromocytoma and Paraganglioma; PRAD, Prostate adenocarcinoma; READ, Rectum adenocarcinoma; SARC, Sarcoma; SKCM, Skin Cutaneous Melanoma; STAD, Stomach adenocarcinoma; TGCT, Testicular Germ Cell Tumors; THCA, Thyroid carcinoma; THYM, Thymoma; UCEC, Uterine Corpus Endometrial Carcinoma; UCS, Uterine Carcinosarcoma.

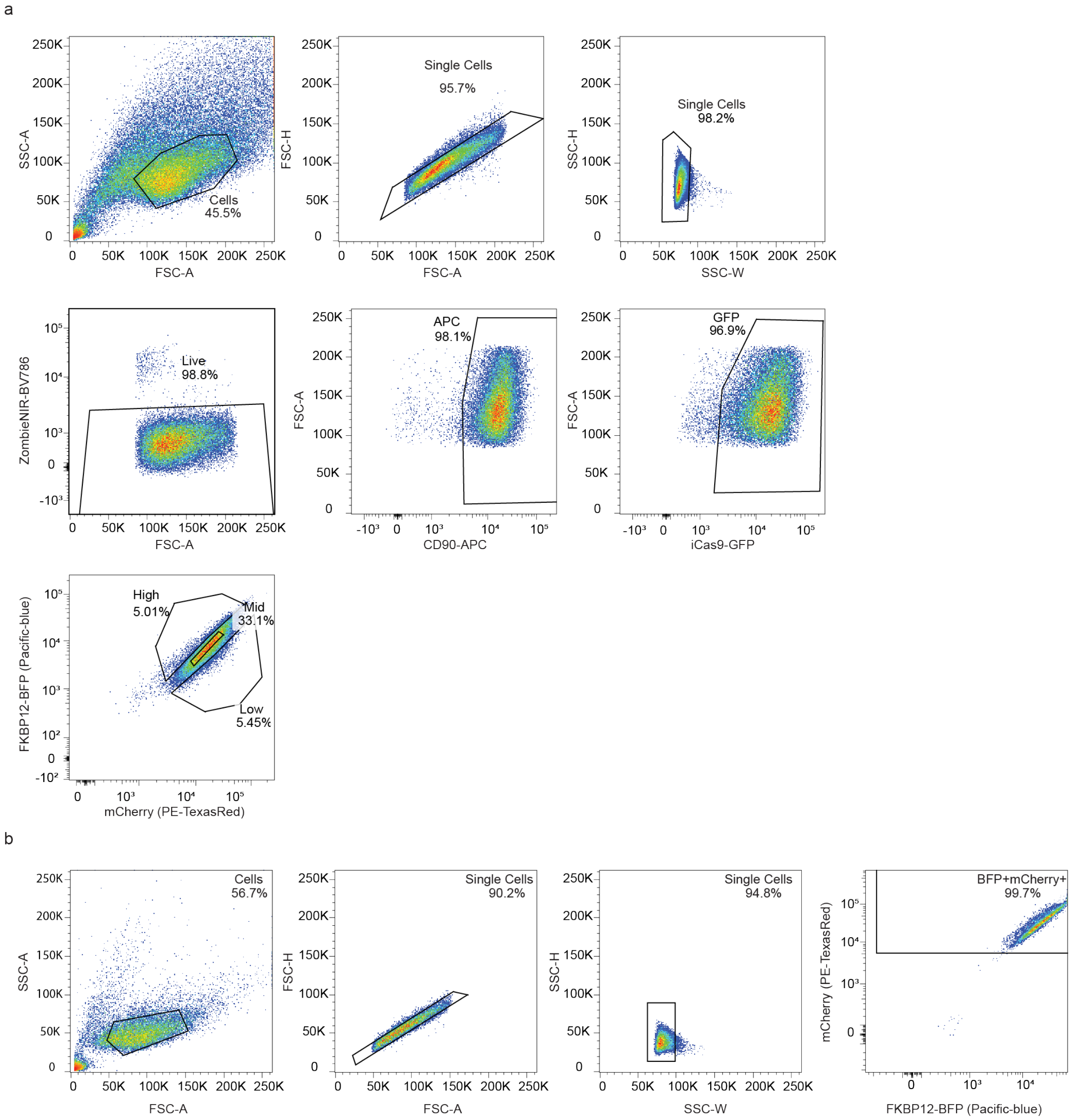

Supplementary Fig. 6 Gating strategy for flow cytometry sorting and analysis.
a Gating strategy for sorting the FACS-based FKBP12-BFP stability CRISPR/iCas9 screen cells (fixed), as in Fig. 2a and Supplementary Fig. 2a. The viable cells were separated from debris using forward scatter area (FSC-A) vs. side scatter area (SSC-A). Single cells were separated from aggregates using FSC-A vs. forward scatter height (FCS-H) and side scatter width (SSC-W) vs. side scatter height (SSC-H). Zombie-NIR staining (BV786) vs FSC-A was used to exclude the dead cells and the CD90-APC$^+$ (sgRNA library) and iCas9-GFP$^+$ cells were sorted into FKBP12^{LOW}, FKBP12^{MID} and FKBP12^{HIGH} based on the FKBP12-BFP (Pacific-blue) vs mCherry (PE-TexasRed) scatter plots. The gates were dynamically adjusted at 5-8% FKBP12^{LOW} and FKBP12^{HIGH} and at 30-35% for FKBP12^{MID}. **b** Gating strategy for all FACS-based degradation assays in (live) cells with the FKBP12-BFP-P2A-mCherry reporter. Live cells were separated from debris and aggregates as described in **a.** The BFP$^+$mCherry$^+$ population was used to compare the BFP/mCherry ratios among different conditions.

Supplementary tables

Supplementary Table 1. Plasmids

Plasmid	Description
pRRL-SFFV-FKBP12-mTagBFP-P2A-mCherry	FKBP12 fluorescent stability reporter
pRRL-SFFV-BRD4s-mTagBFP-P2A-mCherry	BRD4s fluorescent stability reporter (V5-tagged)
pRRL-SFFV-BRD4s-P2A-mCherry	BRD4s without BFP (V5-tagged)
pRRL-U6-sgRNA-EF1αs-Thy1.1-P2A-NeoR	sgRNA cloning vector
pRRL-U6-sg1.FBXO22-IT-EF1αs-Thy1-P2A-NeoR	sgRNA 1 targeting FBXO22
pRRL-U6-sg2.FBXO22-IT-EF1αs-Thy1-P2A-NeoR	sgRNA 2 targeting FBXO22
pRRL-U6-sg3.FBXO22-IT-EF1αs-Thy1-P2A-NeoR	sgRNA 3 targeting FBXO22
pRRL-U6-sg1.SKP1-IT-EF1αs-Thy1-P2A-NeoR	sgRNA 1 targeting SKP1
pRRL-U6-sg1.AAVS1-IT-EF1αs-Thy1-P2A-NeoR	sgRNA 1 targeting AAVS1
pRRL-U6-sg2.AAVS1-IT-EF1αs-Thy1-P2A-NeoR	sgRNA 2 targeting AAVS1
pLEX_puro-P2A-2HA_DEST_N-terminal	2HA cloning vector
pLEX_puro-P2A-HA_FBXO22sgResistant_N-terminal	2HA-FBXO22 cDNA
pLEX_puro-P2A-HA_CRBN_N-terminal	2HA-CRBN cDNA
pLenti6.2-Nanoluc-ccdB	Nanoluciferase cloning vector
pLenti6.2-NLuc-3xFlag-FKBP12	IP experiments/ WB
pLEX-2HA-FBXO22.C143A.sgRes-P2A-puro	2HA-FBXO22 mutant cDNA
pLEX-2HA-FBXO22.C227A.sgRes-P2A-puro	2HA-FBXO22 mutant cDNA
pLEX-2HA-FBXO22.C228A.sgRes-P2A-puro	2HA-FBXO22 mutant cDNA
pLEX-2HA-FBXO22.C326A.sgRes-P2A-puro	2HA-FBXO22 mutant cDNA
pLEX-2HA-FBXO22.C365A.sgRes-P2A-puro	2HA-FBXO22 mutant cDNA
pBiT2.1_SmBiT-FBXO22.WT	NanoBiT assay - WT FBXO22
pBiT2.1_SmBiT-FBXO22.C228A	NanoBiT assay - mutant FBXO22
pBiT2.1_SmBiT-FBXO22.C326A	NanoBiT assay - mutant FBXO22
pBiT1.1-C_LgBiT_FKBP12	NanoBiT assay - FKBP12

Supplementary Table 2. Oligonucleotides & primers

Oligo/primer	5-3 Sequence	Description
FBXO22 sgRNA 1	GCCAGGTTACTCAACACGA	Depletion of FBXO22
FBXO22 sgRNA 2	GCCATGTAAAGAACTGTATG	Depletion of FBXO22
FBXO22 sgRNA 3	GATCCAGGTTACGCTCCGAT	Depletion of FBXO22
SKP1 sgRNA	GTGACTATTAAGACCATGT	Depletion of SKP1
AAVS1 sgRNA 1	GTCACCAATCCTGTCCCTAG	Depletion of AAVS1
AAVS1 sgRNA 2	GGGGCCACTAGGGACAGGAT	Depletion of AAVS1
attB_FBXO22_fw	GGGGACAAGTTTGTACAAAAAAGC<br>AGGCTTAATGGAGCCGGTAGGCTG	Gateway cloning 2HA FBXO22
attB_FBXO22_rv	GGGGACCACTTTGTACAAGAAAGC<br>TGGGTTTTATTTAGATGACCCCAGAT<br>GTATGAGTGC	Gateway cloning 2HA-FBXO22
N-SmBiT-FBXO22_fw	GGTGGTCTCGAGATGGAGCCGGTAG	Restriction enzyme-based cloning of SmBiT-FBXO22
N-SmBiT-FBXO22_rv	AATAATCTCGAGTTATTTAGATGACCCCAGATG	Restriction enzyme-based cloning of SmBiT-FBXO22
Q5_FBXO22-C143A_fw	CCCCAAACAAGCGCAAGTCCTTGGG	FBXO22 cDNA Q5 mutagenesis
Q5_FBXO22-C143A_rv	AATAGCTTCTCAAGGGC	FBXO22 cDNA Q5 mutagenesis
Q5_FBXO22-C227A_fw	TGGTTATAATGCGTGTAAGGTGGGAGC	FBXO22 cDNA Q5 mutagenesis
Q5_FBXO22-C227A_rv	TGGTTATAATGCGTGTAAGGTGGGAGC	FBXO22 cDNA Q5 mutagenesis
Q5_FBXO22-C228A_fw	TTATAATTGCGCGAAGGTGGGAGCC<br>AGTAATTATC	FBXO22 cDNA Q5 mutagenesis
Q5_FBXO22-C228A_rv	CCAAAGACAAGGACC	FBXO22 cDNA Q5 mutagenesis
Q5_FBXO22-C326A_fw	CATGTTTGCAGCGGTTGGCAGGG	FBXO22 cDNA Q5 mutagenesis
Q5_FBXO22-C326A_rv	AAGCCAATGGTGTTATG	FBXO22 cDNA Q5 mutagenesis
Q5_FBXO22-C365A_fw	AGAAATTGGAGCGGATCGGATAGTCACTGG	FBXO22 cDNA Q5 mutagenesis
Q5_FBXO22-C365A_rv	CCATTTCCAAAGAAGCC	FBXO22 cDNA Q5 mutagenesis

Supplementary Methods

Chemistry

General Information

All starting materials, solvents, and reagents used were purchased from commercial sources unless stated otherwise, with no further purification. Reactions were monitored by thin -layer chromatography (TLC) using pre-coated silica gel plates F-254 and visualized using UV light and/or ninhydrin. Proton nuclear magnetic resonance (^{1}H NMR) spectra and carbon nuclear magnetic resonance (^{13}C NMR) spectra were recorded on Bruker AV III 600, AV NEO 600 and AV III HD 700 instruments from the NMR Facility of the University of Vienna. Chemical shifts (δ) are expressed in parts per million (ppm). Data for ^{1}H NMR were denoted as follows: chemical shift, multiplicity (s, singlet; d, doublet; dd, doublet of doublets; t, triplet; m, multiplet; bs, broad singlet) coupling constant (*J*) in Hertz (Hz) and integration. Data for both ^{1}H and ^{13}C NMR spectra were referenced to the corresponding deuterated solvent. Column chromatography was carried out using Biotage Selekt over Biotage Sfar Silica D column cartridges employing Merck silica gel (Kieselgel 60, 63-200 μm). Low resolution mass determinations MS were performed using electrospray ionization (ESI) on Bruker amaZon speed ETD while HR-MS (ESI-TOF) analyses were performed on Bruker timsTOF flex at the MS Facility of the University of Vienna.

SC6N, **SC8N**, **SP2N**, **SP3N**, **JQ1-C6-NH2**, **JQ1-C8-NH2**, **JQ1-P2-NH2**, **JQ1-P3-NH2**, **JQ1-P4-NH2** and **SLF ligand** were provided by WuXi App Tech:

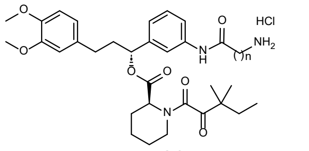

n = 6, **SC6N**
n = 8, **SC8N**

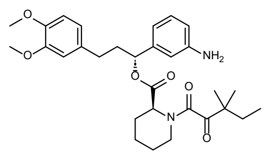

SLF ligand

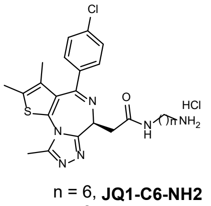

n = 6, **JQ1-C6-NH2**
n = 8, **JQ1-C8-NH2**

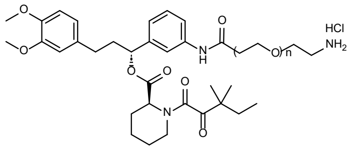

n = 2, **SP2N**
n = 3, **SP3N**

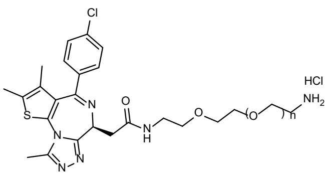

n = 1, JQ1-P2-NH2
n = 2, JQ1-P3-NH2
n = 3, JQ1-P4-NH2

(*R*)-1-(3-(7-aminoheptanamido)phenyl)-3-(3,4-dimethoxyphenyl)propyl (*S*)-1-(3,3-dimethyl-2-oxopentanoyl)piperidine-2-carboxylate (SC6N). ^{1}H NMR (700 MHz, $CDCl_3$) δ 8.89 (bs, 1H), 8.16 (bs, 3H), 7.66 – 7.59 (m, 2H), 7.24 (t, *J* = 8.0 Hz, 1H), 6.99 (d, *J* = 7.4 Hz, 1H), 6.77 – 6.74 (m, 1H), 6.69 – 6.63 (m, 2H), 5.77 (dd, *J* = 7.9, 5.4 Hz, 1H), 5.31 – 5.27 (m, 1H), 3.84 (s, 3H), 3.82 (s, 3H), 3.36 – 3.30 (m, 1H), 3.19 – 3.12 (m, 1H), 3.01 – 2.94 (m, 2H), 2.76 – 2.63 (m, 2H), 2.63 – 2.55 (m, 1H), 2.55 – 2.49 (m, 1H), 2.39 – 2.32 (m, 3H), 2.25 – 2.18 (m, 1H), 2.10 – 2.02 (m, 1H), 1.78 – 1.61 (m, 7H), 1.49 – 1.38 (m, 3H), 1.38 – 1.28 (m, 3H), 1.21 (s, 3H), 1.20 (s, 3H), 0.87 (t, *J* = 7.5 Hz, 3H). ^{13}C NMR (176 MHz, $CDCl_3$) δ 208.4, 172.5, 169.6, 167.2, 148.8, 147.3, 140.7, 138.8, 133.5, 129.1, 121.6, 120.2, 119.5, 117.6, 111.8, 111.3, 77.2, 67.1, 55.9, 55.8, 51.3, 46.7, 44.3, 40.0, 38.8, 38.3, 36.8, 32.5, 31.2, 27.9, 26.8, 26.3, 25.5, 24.9, 23.3, 21.0, 8.7. HRMS (ESI) (m/z) [M+H]$^+$ C37H54N3O7 calc. 652.3956, found 652.3960.

(*R*)-1-(3-(9-aminononanamido)phenyl)-3-(3,4-dimethoxyphenyl)propyl (*S*)-1-(3,3-dimethyl-2-oxopentanoyl)piperidine-2-carboxylate (SC8N). ^{1}H NMR (700 MHz, CDCl3) δ 8.80 (bs, 1H), 8.13 (bs, 3H), 7.69 – 7.64 (m, 1H), 7.60 – 7.55 (m, 1H), 7.26 (t, *J* = 7.9 Hz, 2H), 7.00 (t, *J* = 7.6 Hz, 1H), 6.77 – 6.74 (m, 1H), 6.68 – 6.63 (m, 2H), 5.78 (dd, *J* = 7.9, 5.5 Hz, 1H), 5.31 – 5.27 (m, 1H), 3.84 (s, 3H), 3.83 (s, 3H), 3.35 – 3.29 (m, 1H), 3.29 – 3.21 (m, 2H), 3.15 – 3.09 (m, 1H), 3.00 – 2.93 (m, 2H), 2.62 – 2.49 (m, 2H), 2.43 – 2.32 (m, 3H), 2.25 – 2.17 (m, 1H), 2.09 – 2.02 (m, 1H), 1.77 – 1.60 (m, 7H), 1.48 – 1.42 (m, 1H), 1.40 – 1.25 (m, 8H), 1.21 (s, 6H), 0.88 (t, *J* = 7.5 Hz, 3H). ^{13}C NMR (176 MHz, CDCl3) δ 208.6, 172.7, 169.5, 167.1, 148.8, 147.3, 140.7, 138.7, 133.5, 129.2, 121.8, 120.2, 119.5, 117.6, 111.7, 111.3, 76.7, 55.9, 55.8, 51.3, 46.8, 44.2, 40.0, 38.2, 37.3, 32.5, 31.1, 28.7, 28.5, 28.3, 27.2, 26.2, 26.0, 25.5, 24.9, 23.3, 23.3, 21.0, 8.7. HRMS (ESI) (m/z) [M+H]$^+$ C39H58N3O7 calc. 680.4269, found 680.4262.

(*R*)-1-(3-(3-(2-(2-aminoethoxy)ethoxy)propanamido)phenyl)-3-(3,4-dimethoxyphenyl)propyl(*S*)-1-(3,3-dimethyl-2-oxopentanoyl)piperidine-2-carboxylate (SP2N). ^{1}H NMR (700 MHz, $CDCl_3$) δ 9.40 (bs, 1H), 7.95 (bs, 3H), 7.74 (s, 1H), 7.68 (d, *J* = 7.9 Hz 1H), 7.26 (t, *J* = 67.9 Hz, 1H), 7.00 (d, *J* = 7.7 Hz 1H), 6.79 – 6.74 (m, 1H), 6.70 – 6.63 (m, 2H), 5.73 (dd, *J* = 8.3, 5.2 Hz, 1H), 5.29 – 5.26 (m, 1H), 3.85 (s, 3H), 3.83 (s, 3H), 3.73 – 3.66 (m, 2H), 3.62 – 3.56 (m, 4H), 3.36 – 3.29 (m, 1H), 3.24 – 3.16 (m, 1H), 3.12 – 3.05 (m, 2H), 2.76 – 2.67 (m, 2H), 2.66 – 2.49 (m, 4H), 2.43 – 2.34 (m, 1H), 2.28 – 2.17 (m, 1H), 2.11 – 2.02 (m, 1H), 1.80 – 1.57 (m, 5H), 1.51 – 1.42 (m, 1H), 1.42 – 1.32 (m, 1H), 1.19 (s, 3H), 1.18 (s, 3H), 0.85 (t, *J* = 7.5 Hz, 3H). ^{13}C NMR (176 MHz, $CDCl_3$) δ 208.2, 170.2, 169.8, 167.4, 148.8, 147.3, 140.7, 138.9, 133.5, 129.2, 121.7, 120.2, 119.5, 117.6, 111.7, 111.3, 77.1, 70.2, 69.8, 67.3, 66.3, 55.9, 55.8, 51.5, 46.7, 44.2, 39.6, 38.2, 37.6, 32.4, 31.3, 26.4, 24.9, 23.3, 23.1, 21.1, 8.7. HRMS (ESI) (m/z) [M+H]$^+$ C37H54N3O9 calc. 684.3855, found 684.3846.

(*R*)-1-(3-(3-(2-(2-(2-aminoethoxy)ethoxy)ethoxy)propanamido)phenyl)-3-(3,4-dimethoxyphenyl)propyl (*S*)-1-(3,3-dimethyl-2-oxopentanoyl)piperidine-2-carboxylate (SP3N). ^{1}H NMR (700 MHz, $CDCl_3$) δ 9.57 (bs, 1H), 7.93 (bs, 3H), 7.76 (s, 1H), 7.69 (d, *J* = 7.8 Hz, 1H), 7.26 (t, *J* = 7.9 Hz, 1H), 7.01 (d, *J* = 7.5 Hz, 1H), 6.79 – 6.75 (m, 1H), 6.70 – 6.64 (m, 2H), 5.75 (dd, *J* = 8.2, 5.3 Hz, 1H), 5.33 – 5.29 (m, 1H), 3.85 (s, 3H), 3.84 (s, 3H), 3.75 – 3.71 (m, 2H), 3.64 – 3.60 (m, 6H), 3.58 – 3.54 (m, 2H), 3.37 – 3.32 (m, 1H), 3.22 – 3.17 (m, 1H), 3.10 – 3.03 (m, 2H), 2.84 – 2.77 (m, 2H), 2.64 – 2.58 (m, 1H), 2.58 – 2.51 (m, 1H), 2.44 – 2.34 (m, 3H), 2.26 – 2.19 (m, 1H), 2.11 – 2.03 (m, 1H), 1.80 – 1.59 (m, 5H), 1.53 – 1.43 (m, 1H), 1.43 – 1.32 (m, 2H), 1.21 (s, 3H), 1.20 (s, 3H), 0.87 (t, *J* = 7.5

Hz, 3H). ^{13}C NMR (176 MHz, $CDCl_3$) δ 208.2, 170.5, 169.6, 167.3, 148.8, 147.3, 140.7, 138.9, 133.5, 129.1, 121.7, 120.2, 119.5, 117.6, 111.7, 111.3, 76.9, 70.1, 69.9 (2C), 69.6, 67.3, 66.6, 55.9, 55.8, 51.5, 46.7, 44.2, 39.8, 38.3, 37.7, 32.4, 31.3, 26.4, 24.9, 23.4, 23.2, 21.1, 8.7. HRMS (ESI) (m/z) $[M+H]^+$ C39H58N3O10 calc. 728.4117, found 728.4102.

(*S*)-*N*-(6-aminohexyl)-2-(4-(4-chlorophenyl)-2,3,9-trimethyl-6*H*-thieno[3,2-f][1,2,4]triazolo[4,3-a][1,4]diazepin-6-yl)acetamide (JQ1-C6-NH2). ^{1}H NMR (700 MHz, $CDCl_3$) δ 8.14 (bs, 3H), 8.06 (bs, 1H), 7.53 – 7.43 (m, 2H), 7.36 – 7.33 (m, 2H), 4.84 (t, *J* = 7.0 Hz, 1H), 3.67 – 3.59 (m, 1H), 3.58 – 3.50 (m, 1H), 3.36 – 3.29 (m, 1H), 3.20 – 3.13 (m, 1H), 2.98 – 2.90 (m, 2H), 2.85 (s, 3H), 2.41 (s, 3H), 1.76 – 1.68 (m, 2H), 1.63 (s, 3H), 1.57 – 1.49 (m, 2H), 1.43 – 1.32 (m, 4H).^{13}C NMR (176 MHz, $CDCl_3$) δ 169.6, 165.9, 154.7, 151.0, 138.1, 134.4, 133.1, 131.9, 131.6, 131.0, 130.4, 128.9, 53.3, 39.5, 39.1, 36.9, 28.3, 26.7, 25.6, 25.4, 14.4, 13.3, 11.9. HRMS (ESI) (m/z) $[M+H]^+$ C25H32ClN6OS, calc. 499.2041, found 499.2050.

(*S*)-*N*-(8-aminooctyl)-2-(4-(4-chlorophenyl)-2,3,9-trimethyl-6*H*-thieno[3,2-f][1,2,4]triazolo[4,3-a][1,4]diazepin-6-yl)acetamide (JQ1-C8-NH2). ^{1}H NMR (600 MHz, $CDCl_3$) δ 8.09 (bs, 3H), 7.90 (bs, 1H), 7.55 – 7.46 (m, 2H), 7.39 – 7.34 (m, 2H), 4.87 (t, *J* = 7.0 Hz, 1H), 3.68 – 3.56 (m, 2H), 3.31 – 3.22 (m, 2H), 2.92 – 2.86 (m, 2H), 2.82 (s, 3H), 2.41 (s, 3H), 1.72 – 1.64 (m, 2H), 1.63 (s, 3H), 1.56 – 1.49 (m, 2H), 1.39 – 1.33 (m, 4H), 1.32 – 1.28 (m, 4H). ^{13}C NMR (151 MHz, CDCl3) δ 169.4, 166.5, 154.4, 151.1, 138.6, 133.7, 133.1, 132.8, 131.7, 131.4, 129.7, 128.9, 53.1, 39.9, 39.4, 36.4, 28.7, 27.9, 27.9, 26.7, 25.9, 25.4, 14.4, 13.2, 11.8. HRMS (ESI) (m/z) $[M+H]^+$ C27H36ClN6OS, calc. 527.2354, found 527.2349.

(*S*)-*N*-(2-(2-(2-aminoethoxy)ethoxy)ethyl)-2-(4-(4-chlorophenyl)-2,3,9-trimethyl-6*H*-thieno[3,2-f][1,2,4]triazolo[4,3-a][1,4]diazepin-6-yl)acetamide (JQ1-P2-NH2). ^{1}H NMR (600 MHz, $CDCl_3$) δ 8.51 (bs, 1H), 8.09 (s, 3H), 7.64 – 7.57 (m, 2H), 7.40 – 7.37 (m, 2H), 5.12 – 5.06 (m, 1H), 3.82 – 3.61 (m, 9H), 3.53 – 3.43 (m, 3H), 3.19 – 3.10 (m, 2H), 3.00 (s, 3H), 2.42 (s, 3H), 1.60 (s, 3H). ^{13}C NMR (151 MHz, $CDCl_3$) δ 169.4, 167.0, 153.8, 152.2, 140.9, 139.8, 135.6, 134.6, 133.5, 132.4, 131.3, 129.0, 70.0 (2C), 69.5, 66.5, 52.3, 39.8, 39.6, 34.8, 14.4, 13.4, 12.1. HRMS (ESI) (m/z) [M+H] C25H32ClN6O3S, calc. 531.1940, found 531.1950.

(*S*)-*N*-(2-(2-(2-(2-aminoethoxy)ethoxy)ethoxy)ethyl)-2-(4-(4-chlorophenyl)-2,3,9-trimethyl-6*H*-thieno[3,2-f][1,2,4]triazolo[4,3-a][1,4]diazepin-6-yl)acetamide (JQ1-P3-NH2). ^{1}H NMR (600 MHz, $CDCl_3$) δ 8.56 (bs, 1H), 8.12 (bs, 3H), 7.70 – 7.58 (m, 2H), 7.44 – 7.35 (m, 2H), 5.21 – 5.08 (m, 1H), 3.91 – 3.82 (m, 1H), 3.81 – 3.70 (m, 3H), 3.70 – 3.59 (m, 10H), 3.56 – 3.40 (m, 2H), 3.12 – 3.03 (m, 2H), 2.98 (s, 3H), 2.42 (s, 3H), 1.57 (s, 3H). ^{13}C NMR (151 MHz, $CDCl_3$) δ 169.2, 166.9, 153.6, 152.6, 140.6, 135.2, 134.6, 133.1, 132.8, 130.6, 129.2, 128.4, 70.5, 70.2, 69.8 (2C), 69.7, 66.6, 52.1, 39.8, 39.6, 34.2, 14.7, 13.6, 12.2. HRMS (ESI) (m/z) $[M+H]^+$ C27H36ClN6O4S calc. 575.2202, found 575.2213.

(*S*)-*N*-(14-amino-3,6,9,12-tetraoxatetradecyl)-2-(4-(4-chlorophenyl)-2,3,9-trimethyl-6*H*-thieno[3,2-f][1,2,4]triazolo[4,3-a][1,4]diazepin-6-yl)acetamide (JQ1-P4-NH2). ^{1}H NMR (600 MHz, $CDCl_3$) δ 8.68 (bs, 1H), 7.97 (bs, 3H), 7.65 – 7.56 (m, 2H), 7.40 – 7.32 (m, 2H), 5.35 – 5.27 (m, 1H), 3.92 – 3.85 (m, 1H), 3.85 – 3.75 (m, 3H), 3.71 – 3.59 (m, 14H), 3.58 – 3.48 (m, 1H), 3.46 – 3.38 (m, 1H), 3.16 – 3.05 (m, 5H), 2.42 (s, 3H), 1.49 (s, 3H). ^{13}C NMR (151 MHz, $CDCl_3$) δ 168.8, 167.0, 153.2, 153.0, 141.0, 135.5, 135.2, 133.6, 133.1, 129.0, 128.8, 127.7, 70.2, 70.1, 70.1, 70.0, 70.0, 69.9, 69.8, 66.8, 51.4, 40.0, 39.3, 33.2, 14.3, 13.5, 12.3. HRMS (ESI) (m/z) $[M+H]^+$ C29H40ClN6O5S calc. 619.2464, found 619.2460.

Synthesis of SP3NAc

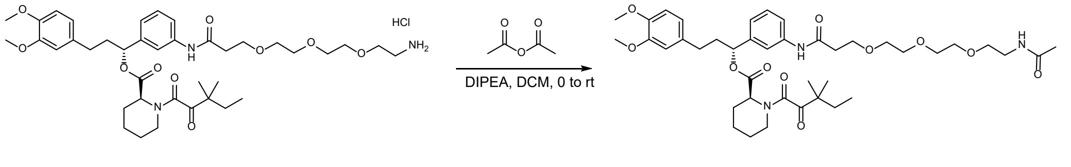

SP3N **SP3NAc**

To a solution of **SP3N** hydrochloride salt (20 mg, 0.026 mmol) in dichloromethane (0.5 mL), acetic anhydride (3.7 uL, 1.5 eq) is added at 0 C and the reaction stirred at room temperature for 1 h until no starting material is observed. The mixture is diluted with dichloromethane and washed with water, 1.0 N HCl and brine. The organic layer is dried over anhydrous Na_2SO_4, filtered and concentrated under

reduced pressure. The desired compound is purified by flash-chromatography using dichloromethane/methanol.

(*R*)-3-(3,4-dimethoxyphenyl)-1-(3-(2-oxo-6,9,12-trioxa-3-azapentadecan-15-amido)phenyl)propyl (*S*)-1-(3,3-dimethyl-2-oxopentanoyl)piperidine-2-carboxylate (SP3NAc). 12 mg. 60%. White powder. ^{1}H NMR (600 MHz, $CDCl_3$) δ 8.65 (bs, 1H), 7.58 (bs, 1H), 7.57 – 7.53 (m, 1H), 7.28 (t, *J* = 7.8 Hz, 1H), 7.03 (d, *J* = 7.5 Hz, 1H), 6.80 – 6.74 (m, 1H), 6.72 – 6.64 (m, 2H), 6.19 (s, 1H), 5.78 (dd, *J* = 8.0, 5.5 Hz, 1H), 5.32 – 5.29 (m, 1H), 3.88 – 3.82 (m, 8H), 3.72 – 3.66 (m, 4H), 3.65 – 3.61 (m, 2H), 3.58 – 3.54 (m, 2H), 3.48 (t, *J* = 5.1 Hz, 2H), 3.40 – 3.31 (m, 3H), 3.21 – 3.11 (m, 1H), 2.65 (t, *J* = 5.8 Hz, 2H), 2.62 – 2.57 (m, 1H), 2.57 – 2.49 (m, 1H), 2.38 – 2.33 (m, 1H), 2.29 – 2.19 (m, 1H), 2.10 – 2.03 (m, 1H), 1.94 (s, 3H), 1.78 – 1.59 (m, 5H), 1.51 – 1.42 (m, 1H), 1.41 – 1.31 (m, 2H), 1.23 (s, 2H), 1.22 (s, 3H), 0.89 (t, *J* = 7.5 Hz, 3H). ^{13}C NMR (176 MHz, $CDCl_3$) δ 208.4, 170.5, 170.0, 169.7, 167.3, 149.0, 147.4, 140.9, 138.8, 133.6, 129.2, 122.0, 120.3, 119.7, 117.9, 111.9, 111.4, 76.8, 70.6, 70.4 (2C), 70.2, 69.9, 67.2, 56.1, 56.0, 51.4, 46.9, 44.3, 39.5, 38.3, 38.1, 32.6, 31.3, 26.5, 25.1, 23.5, 23.4, 23.3, 21.2, 8.9. HRMS (ESI) (m/z) $[M+Na]^+$ C41H59N3O11 calc. 792.4042, found 792.4035.

Synthesis of aldehydes SP2CHO-SP3CHO and protected aldehyde SP2CHOp.

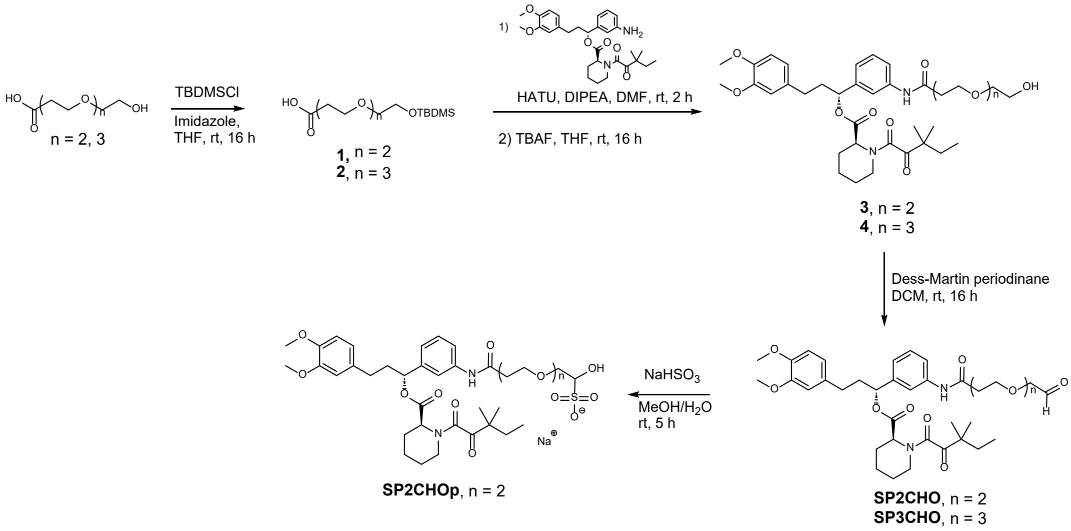

Synthesis of carboxylic acids **1-2**

To a solution of hydroxy carboxylic acid (100 mg) in THF (0.5 M), imidazole (1.4 eq.) is added and stirred for 30 min at room temperature. TBDMSCl (1.4 eq.) is added and the reaction stirred at room temperature for 16 h until no starting material is observed. The mixture is diluted with ethyl acetate and washed with water and brine. The organic layer is dried over anhydrous Na_2SO_4, filtered, and concentrated under reduced pressure. The resulting compounds are used for the next step without further purification.

2,2,3,3-tetramethyl-4,7,10-trioxa-3-silatridecan-13-oic acid (1). 120 mg. 87%. Colorless oil. ^{1}H NMR (600 MHz, $CDCl_3$) δ 3.75 (t, *J* = 5.2 Hz, 2H), 3.67 – 3.63 (m, 4H), 3.62 – 3.59 (m, 2H), 3.56 (d, *J* = 5.1 Hz, 2H), 2.58 (t, *J* = 3.4 Hz, 2H), 0.87 (s, 9H), 0.05 (s, 6H). ^{13}C NMR (151 MHz, CDCl3) δ 177.2, 70.2, 69.8, 67.3, 66.3, 62.6, 36.8, 25.9, 18.1, -4.7, -4.8. MS (ESI) (m/z) $[M-H]^-$ C13H27O5Si calc. 291.17, found 291.16.

2,2,3,3-tetramethyl-4,7,10,13-tetraoxa-3-silahexadecan-16-oic acid (2). 150 mg. 99%. Colorless oil. ^{1}H NMR (600 MHz, $CDCl_3$) δ 3.80 (t, *J* = 5.2 Hz, 2H), 3.72 – 3.68 (m, 2H), 3.67 – 3.60 (m, 8 H), 3.56 (t, *J* = 5.1 Hz, 2H), 2.63 (t, *J* = 3.4 Hz, 2H), 0.89 (s, 9H), 0.07 (s, 6H). MS (ESI) (m/z) $[M-H]^-$ C15H31O6Si calc. 335.20, found 335.19.

Synthesis of alcohols **3-4**.

A mixture of carboxylic acids **1** or **2** (1.4 eq.), SLF ligand (1.0 eq.), HATU (1.9 eq.) and DIPEA (1.0 eq) in DMF (0.5 M) is stirred at room temperature until completion for 2 hours. The reaction is diluted with ethyl acetate and washed with water, sat. NH_4Cl and brine. The organic layer is dried over anhydrous Na_2SO_4, filtered and concentrated under reduced pressure. After purification by flash chromatography using hexane/ethyl acetate the resulting compound is dissolved in THF (0.2 M) and 1.0 M solution of

TBAF in THF (1.5 eq) is added dropwise at 0 C. The reaction is allowed to warm to room temperature and stirred for 16 hours until no starting material is observed. The reaction is quenched with water and extracted with ethyl acetate. Organic layer is dried over Na_2SO_4, filtered and concentrated under reduced pressure. Desired compounds are used for the next step without further purification.

(*R*)-3-(3,4-dimethoxyphenyl)-1-(3-(3-(2-(2-hydroxyethoxy)ethoxy)propanamido)phenyl)propyl (*S*)-1-(3,3-dimethyl-2-oxopentanoyl)piperidine-2-carboxylate (3). Yellow oil. ^{1}H NMR (600 MHz, $CDCl_3$) δ 8.61 (bs, 1H), 7.63 (d, *J* = 8.0 Hz, 1H), 7.51 (s, 1H), 7.30 (t, *J* = 7.9 Hz, 1H), 7.04 (d, *J* = 7.6 Hz, 1H), 6.80 – 6.75 (m, 1H), 6.70 – 6.64 (m, 2H), 5.78 (dd, *J* = 7.9, 5.6 Hz, 1H), 5.33 – 5.30 (m, 1H), 3.87 – 3.82 (m, 8H), 3.72 – 3.66 (m, 6H), 3.63 – 3.58 (m, 2H), 3.38 – 3.31 (m, 1H), 3.18 – 3.11 (m, 1H), 2.66 (t, *J* = 5.5 Hz, 2H), 2.63 – 2.57 (m, 1H), 2.57 – 2.50 (m, 1H), 2.39 – 2.33 (m, 1H), 2.28 – 2.20 (m, 1H), 2.09 – 2.04 (m, 1H), 1.76 – 1.60 (m, 5H), 1.52 – 1.42 (m, 1H), 1.41 – 1.32 (m, 1H), 1.23 (s, 3H), 1.22 (s, 3H), 0.89 (t, *J* = 7.5 Hz, 3H). ^{13}C NMR (176 MHz, $CDCl_3$) δ 208.2, 170.2, 169.7, 167.3, 148.8, 147.3, 140.7, 138.9, 133.5, 129.1, 121.6, 120.2, 119.5, 117.7, 111.7, 111.3, 77.1, 70.0, 69.2, 67.3, 66.3, 60.3, 55.9, 55.8, 51.5, 46.7, 44.2, 38.3, 37.4, 32.4, 31.3, 26.4, 24.9, 23.3, 23.1, 21.1, 8.7. MS (ESI) (m/z) $[M+Na]^+$ C37H52N2O10 calc. 707.36, found 707.48.

(*R*)-3-(3,4-dimethoxyphenyl)-1-(3-(3-(2-(2-(2-hydroxyethoxy)ethoxy)ethoxy)propanamido)phenyl)propyl (*S*)-1-(3,3-dimethyl-2-oxopentanoyl)piperidine-2-carboxylate (4). Yellow oil. ^{1}H NMR (600 MHz, $CDCl_3$) δ 8.81 (bs, 1H), 7.63 (s, 1H), 7.52 (d, *J* = 8.0 Hz, 1H), 7.28 (t, *J* = 7.8 Hz, 1H), 7.03 (d, *J* = 7.8 Hz, 1H), 6.79 – 6.75 (m, 1H), 6.71 – 6.64 (m, 2H), 5.78 (dd, *J* = 8.0, 5.6 Hz, 1H), 5.32 – 5.29 (m, 1H), 3.85 (s, 3H), 3.84 (s, 3H), 3.82 (t, *J* = 5.5 Hz, 2H), 3.71 – 3.68 (m, 2H), 3.68 – 3.66 (m, 4H), 3.65 – 3.61 (m, 4H), 3.58 – 3.56 (m, 2H), 3.38 – 3.31 (m, 1H), 3.18 – 3.14 (m, 1H), 2.64 (t, *J* = 5.8 Hz, 2H), 2.62 – 2.57 (m, 1H), 2.57 – 2.48 (m, 1H), 2.39 – 2.32 (m, 1H), 2.29 – 2.20 (m, 1H), 2.11 – 2.03 (m, 1H), 1.77 – 1.64 (m, 5H), 1.49 – 1.44 (m, 1H), 1.38 – 1.32 (m, 1H), 1.23 (s, 3H), 1.21 (s, 3H), 0.89 (t, *J* = 7.4 Hz, 3H). ^{13}C NMR (176 MHz, $CDCl_3$) δ 208.2, 170.1, 169.6, 167.2, 148.8, 147.3, 140.7, 138.8, 133.5, 129.1, 121.8, 120.2, 119.6, 117.7, 111.7, 111.3, 76.8, 69.9 (2C), 69.6, 68.7, 67.3, 66.6, 60.2, 55.9, 55.8, 51.5, 46.7, 44.2, 38.3, 37.9, 32.4, 31.3, 26.4, 24.9, 23.4, 23.2, 21.1, 8.7. MS (ESI) (m/z) $[M+Na]^+$ C39H56N2O11 calc. 751.38, found 751.50.

Synthesis of aldehydes **SP2CHO** and **SP3CHO**

To a solution of alcohols **3** or **4** (1.0 eq) in dichloromethane (0.1 M), Dess Martin periodinane (1.2 eq.) is added portion wise at room temperature. The reaction slowly turns red and is stirred until no alcohol is observed in TLC (around 2-3 h). 1 mL of 10% $Na_2S_2O_3$ is added and stirred for 30 minutes, followed by dilution with dichloromethane and separation of layer. Organic layer is washed with water and brine, dried over Na_2SO_4, filtered and concentrated under reduced pressure. Desired compounds are purified by flash chromatography using ethyl acetate/methanol as solvents. Since **SP2CHO** was not pure enough according to NMR, we proceed to its transformation into the corresponding hydroxysulfonate sodium salt.

(*R*)-3-(3,4-dimethoxyphenyl)-1-(3-(3-(2-(2-oxoethoxy)ethoxy)propanamido)phenyl)propyl (*S*)-1-(3,3-dimethyl-2-oxopentanoyl)piperidine-2-carboxylate (SP2CHO). 50 mg. 85%. Dark yellow oil. ^{1}H NMR (600 MHz, $CDCl_3$) δ 9.65 (bs, 1H), 8.51 (bs, 1H), 7.59 (d, 1H), 7.51 (s, 1H), 7.29 (t, *J* = 8.0 Hz 1H), 7.03 (d, *J* = 7.7 Hz, 1H), 6.79 – 6.76 (m, 1H), 6.69 – 6.66 (m, 2H), 5.76 (dd, *J* = 8.0, 5.0 Hz, 1H), 5.33 – 5.30 (m, 1H), 4.16 (d, *J* = 2.9 Hz, 2H), 3.88 – 3.80 (m, 8H), 3.77 – 3.74 (m, 2H), 3.73 – 3.68 (m, 2H), 3.36 – 3.31 (m, 1H), 3.17 – 3.10 (m, 1H), 2.66 (t, *J* = 5.6 Hz, 2H), 2.64 – 2.56 (m, 1H), 2.56 – 2.51 (m, 1H), 2.38 – 2.32 (m, 1H), 2.27 – 2.19 (m, 1H), 2.09 – 2.02 (m, 1H), 1.77 – 1.58 (m, 5H), 1.48 – 1.42 (m, 1H), 1.41 – 1.31 (m, 1H), 1.23 (s, 3H), 1.22 (s, 3H), 0.89 (t, *J* = 7.5 Hz, 3H). MS (ESI) (m/z) $[M+Na]^+$ C37H50N2O10 calc. 705.33, found 705.50.

(*R*)-3-(3,4-dimethoxyphenyl)-1-(3-(3-(2-(2-(2-oxoethoxy)ethoxy)ethoxy)propanamido)phenyl)propyl (*S*)-1-(3,3-dimethyl-2-oxopentanoyl)piperidine-2-carboxylate (SP3CHO). 26 mg. 42%. Yellow oil. ^{1}H NMR (600 MHz, $CDCl_3$) δ 9.65 (bs, 1H), 8.64 (bs, 1H), 7.60 (s, 1H), 7.51 (d, *J* = 8.4 Hz, 1H), 7.28 (t, *J* = 7.8 Hz, 1H), 7.04 (d, *J* = 7.5 Hz, 1H), 6.79 – 6.75 (m, 1H), 6.69 – 6.65 (m, 2H), 5.78 (dd, *J* = 8.0, 5.4 Hz, 1H), 5.34 – 5.30 (m, 1H), 4.08 (s, 2H), 3.88 – 3.81 (m, 8H), 3.72 – 3.63 (m, 8H), 3.38 – 3.32 (m, 1H), 3.19 – 3.14 (m, 1H), 2.67 (t, *J* = 5.7 Hz, 2H), 2.62 – 2.57 (m, 1H), 2.57 – 2.51 (m, 1H), 2.39 – 2.33 (m, 1H), 2.26 – 2.20 (m, 1H), 2.10 – 2.06 (m, 1H), 1.77 – 1.61 (m, 5H), 1.51 – 1.42 (m, 1H), 1.41 – 1.33 (m, 1H), 1.23 (s, 3H), 1.22 (s, 3H), 0.89 (t, *J* = 7.5 Hz, 3H). ^{13}C NMR (176 MHz, $CDCl_3$) δ 208.2, 200.8, 169.9, 169.6, 167.1, 148.9, 147.3, 140.7, 138.7, 133.5, 129.1, 121.9, 120.1, 119.6, 117.9, 111.7, 111.3, 76.7, 76.7,

71.1, 70.6, 70.4, 70.2, 67.0, 55.9, 55.8, 51.2, 46.7, 44.2, 38.2, 38.0, 32.5, 31.2, 26.3, 25.0, 23.4, 23.2, 21.1, 8.7. HRMS (ESI) (m/z) $[M+Na]^+$ C39H54N2O11 calc. 749.3620, found 749.3606.

Synthesis of hydroxysulfonate salt **SP2CHOp**.

To a solution of **SP2CHO** (50 mg, 0.07 mmol) in methanol (0.14 mL) 0.02 mL of 3.3 M solution of $NaHSO_3$ is added dropwise at room temperature. The reaction is stirred for 5 hours until no starting material is observed by TLC. Solvent is evaporated under reduced pressure and the residue is washed with diethyl ether and dichloromethane/ether (1:1). The resulting pale-yellow solid is collected and dried.

Sodium 2-(2-(3-((3-((*R*)-3-(3,4-dimethoxyphenyl)-1-(((*S*)-1-(3,3-dimethyl-2-oxopentanoyl)piperidine-2-carbonyl)oxy)propyl)phenyl)amino)-3-oxopropoxy)ethoxy)-1-hydroxyethane-1-sulfonate (SP2CHOp). 30 mg. 54%. Pale yellow powder. ^{1}H NMR (600 MHz, DMSO) δ 10.01 (s, 1H), 7.75 – 7.70 (m, 1H), 7.47 (d, *J* = 7.5 Hz 1H), 7.29 (t, *J* = 7.4 Hz, 1H), 7.01 (d, *J* = 7.8 Hz, 1H), 6.85 (d, *J* = 8.2 Hz, 1H), 6.80 – 6.73 (m, 1H), 6.68 (d, *J* = 7.9 Hz, 1H), 5.63 (dd, *J* = 8.7, 4.8 Hz, 1H), 5.53 – 5.46 (m, 1H), 5.17 – 5.09 (m, 1H), 4.06 – 3.99 (m, 1H), 3.78 (d, *J* = 10.0 Hz, 1H), 3.75 – 3.66 (m, 7H), 3.52 – 3.26 (m, 6H), 3.19 – 3.15 (m, 1H), 2.57 – 2.49 (m, 6H), 2.25 – 2.19 (m, 1H), 2.14 – 2.10 (m, 1H), 2.04 – 1.99 (m, 1H), 1.73 – 1.54 (m, 5H), 1.37 – 1.31 (m, 1H), 1.24 – 1.19 (m, 1H), 1.16 (s, 3H), 1.14 (s, 3H), 0.80 (t, *J* = 7.4 Hz, 3H). ^{13}C NMR (176 MHz, $CDCl_3$) δ 208.0, 170.1, 169.7, 167.3, 148.8, 147.3, 140.7, 138.9, 133.5, 129.0, 121.5, 120.2, 119.4, 117.7, 111.8, 111.3, 81.7, 77.1, 70.2, 69.5, 67.3, 67.1, 55.9, 55.8, 51.4, 46.7, 44.2, 38.3, 37.3, 32.4, 31.3, 26.5, 24.9, 23.4, 23.1, 21.2, 8.7. HRMS (ESI) (m/z) [M+Na] C37H52N2O13S (sulfonic acid) calc. 787.3082, found 787.3087.

Synthesis of SP2-Cl, SP2-Acry, SP3-Cl and SP3-Acry.

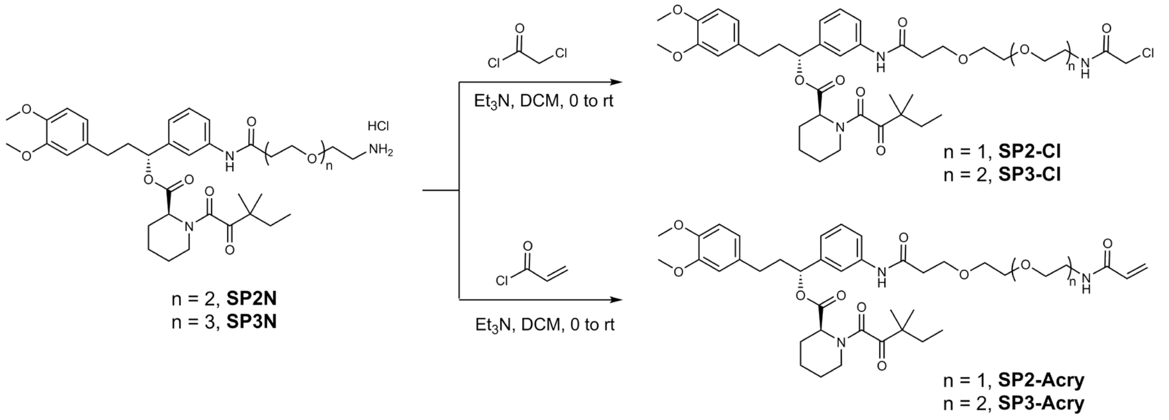

To a solution of **SP2N** or **SP3N** hydrochloride salt (10-20 mg) in dichloromethane (0.1 M), triethylamine (2.5 eq) is added at 0 C and the mixture stirred at 0 C for 30 min. Corresponding acid chloride (1.2 eq) is added and the reaction stirred at room temperature for 2-3 h until no starting material is observed. The mixture is diluted with dichloromethane and washed with water, 1.0 N HCl and brine. The organic layer is dried over anhydrous Na_2SO_4, filtered and concentrated under reduced pressure. The desired compound is purified by flash-chromatography using dichloromethane/methanol.

(*R*)-1-(3-(3-(2-(2-(2-chloroacetamido)ethoxy)ethoxy)propanamido)phenyl)-3-(3,4-dimethoxyphenyl)propyl (*S*)-1-(3,3-dimethyl-2-oxopentanoyl)piperidine-2-carboxylate (SP2-Cl). 15 mg. 70%. White powder. ^{1}H NMR (600 MHz, $CDCl_3$) δ 8.53 (bs, 1H), 7.58 (d, *J* = 8.4 Hz, 1H), 7.52 (s, 1H), 7.29 (t, *J* = 8.0 Hz, 1H), 7.04 (d, *J* = 7.6 Hz, 1H), 6.96 (bs, 1H), 6.79 – 6.75 (m, 1H), 6.70 – 6.65 (m, 2H), 5.77 (dd, *J* = 8.0, 5.5 Hz, 1H), 5.33 – 5.30 (m, 1H), 3.98 (s, 2H), 3.86 (s, 3H), 3.84 (s, 3H), 3.74 – 3.65 (m, 6H), 3.59 (t, *J* = 5.3 Hz, 2H), 3.48 – 3.44 (m, 2H), 3.37 – 3.32 (m, 1H), 3.17 – 3.11 (m, 1H), 2.66 (t, *J* = 5.7 Hz, 2H), 2.63 – 2.57 (m, 1H), 2.57 – 2.51 (m, 1H), 2.38 – 2.33 (m, 1H), 2.28 – 2.19 (m, 1H), 2.10 – 2.03 (m, 1H), 1.77 – 1.60 (m, 5H), 1.50 – 1.42 (m, 1H), 1.41 – 1.33 (m, 1H), 1.23 (s, 3H), 1.22 (s, 3H), 0.89 (t, *J* = 7.5 Hz, 3H). ^{13}C NMR (151 MHz, $CDCl_3$) δ 207.32, 168.77, 168.65, 166.08, 165.14, 147.86, 146.30, 139.79, 137.59, 132.45, 128.19, 120.89, 119.15, 118.47, 116.81, 110.73, 110.28, 75.62, 69.23, 69.03, 68.38, 65.96, 54.91, 54.83, 50.21, 45.74, 43.22, 41.60, 38.36, 37.15, 36.95, 31.49, 30.15, 25.27, 23.96, 22.36, 22.25, 20.03, 7.75. HRMS (ESI) (m/z) $[M+Na]^+$ C39H54ClN3O10 calc. 782.3390, found 782.3383.

(*R*)-1-(3-(3-(2-(2-acrylamidoethoxy)ethoxy)propanamido)phenyl)-3-(3,4-dimethoxyphenyl)propyl (*S*)-1-(3,3-dimethyl-2-oxopentanoyl)piperidine-2-carboxylate (SP2-Acry). 12 mg. 58%. White

powder. ^{1}H NMR (600 MHz, $CDCl_3$) δ 8.61 (bs, 1H), 7.65 (d, *J* = 8.2 Hz, 1H), 7.47 (s, 1H), 7.29 (t, *J* = 8.0 Hz, 1H), 7.03 (d, *J* = 7.7 Hz, 1H), 6.78 – 6.75 (m, 1H), 6.69 – 6.65 (m, 2H), 6.27 (bs, 1H), 6.23 (dd, *J* = 17.0, 1.5 Hz, 1H), 6.03 (dd, *J* = 17.0, 10.0 Hz, 1H), 5.76 (dd, *J* = 7.9, 5.5 Hz, 1H), 5.56 (dd, *J* = 10.3, 1.5 Hz, 1H), 5.33 – 5.29 (m, 1H), 3.86 (s, 3H), 3.84 (s, 3H), 3.72 (q, *J* = 7.0 Hz, 2H), 3.69 – 3.64 (m, 4H), 3.58 (t, *J* = 5.2 Hz, 2H), 3.51 – 3.43 (m, 2H), 3.38 – 3.31 (m, 1H), 3.18 – 3.09 (m, 1H), 2.65 (t, *J* = 5.7 Hz, 2H), 2.63 – 2.58 (m, 1H), 2.58 – 2.52 (m, 1H), 2.38 – 2.33 (m, 1H), 2.28 – 2.20 (m, 1H), 2.10 – 2.03 (m, 1H), 1.77 – 1.60 (m, 5H), 1.49 – 1.42 (m, 1H), 1.40 – 1.33 (m, 1H), 1.23 (s, 3H), 1.22 (s, 3H), 0.89 (t, *J* = 7.5 Hz, 3H). ^{13}C NMR (151 MHz, $CDCl_3$) δ 207.3, 168.9, 168.7, 166.1, 164.7, 147.9, 146.3, 139.7, 137.6, 132.4, 129.7, 128.2, 125.4, 120.8, 119.2, 118.6, 116.8, 110.7, 110.3, 75.6, 69.2, 68.9, 68.7, 65.8, 54.9, 54.8, 50.2, 45.7, 43.2, 38.0, 37.1, 36.9, 31.5, 30.1, 25.3, 23.9, 22.3, 22.3, 20.0, 7.7. HRMS (ESI) (m/z) $[M+Na]^+$ C40H55N3O10 calc. 760.3780, found 760.3769.

(*R*)-1-(3-(1-chloro-2-oxo-6,9,12-trioxa-3-azapentadecan-15-amido)phenyl)-3-(3,4-dimethoxyphenyl)propyl (*S*)-1-(3,3-dimethyl-2-oxopentanoyl)piperidine-2-carboxylate (SP3-Cl). 7 mg. 68%. ^{1}H NMR (600 MHz, $CDCl_3$) δ 8.59 (bs, 1H), 7.60 (s, 1H), 7.52 (d, *J* = 8.0 Hz, 1H), 7.29 (t, *J* = 7.8 Hz, 1H), 7.05 (d, *J* = 7.4 Hz, 1H), 6.98 (bs, 1H), 6.81 – 6.75 (m, 1H), 6.75 – 6.62 (m, 2H), 5.78 (dd, *J* = 7.8, 5.6 Hz, 1H), 5.35 – 5.27 (m, 1H), 4.02 (s, 2H), 3.88 – 3.80 (m, 8H), 3.73 – 3.66 (m, 4H), 3.65 – 3.62 (m, 2H), 3.61 – 3.56 (m, 2H), 3.56 – 3.50 (m, 2H), 3.48 – 3.43 (m, 2H), 3.39 – 3.32 (m, 1H), 3.22 – 3.13 (m, 1H), 2.68 – 2.62 (m, 2H), 2.62 – 2.57 (m, 1H), 2.57 – 2.51 (m, 1H), 2.39 – 2.34 (m, 1H), 2.27 – 2.19 (m, 1H), 2.11 – 2.03 (m, 1H), 1.77 – 1.71 (m, 5H), 1.51 – 1.42 (m, 1H), 1.41 – 1.32 (m, 1H), 1.24 (s, 3H), 1.22 (s, 3H), 0.89 (t, *J* = 7.5 Hz, 3H). ^{13}C NMR (151 MHz, $CDCl_3$) δ 208.2, 169.9, 169.6, 167.1, 166.0, 148.9, 147.3, 140.8, 138.7, 133.5, 129.1, 121.9, 120.2, 119.6, 117.9, 111.8, 111.3, 76.7, 70.5, 70.4, 70.3 (2C), 69.3, 67.1, 55.9, 55.8, 51.2, 46.7, 44.2, 42.6, 39.6, 38.2, 38.0, 32.5, 31.2, 26.3, 25.0, 23.4, 23.2, 21.1, 8.7. HRMS (ESI) (m/z) $[M+Na]^+$ C41H58ClN3O11 calc. 826.3652, found 826.3660.

(*R*)-3-(3,4-dimethoxyphenyl)-1-(3-(3-oxo-7,10,13-trioxa-4-azahexadec-1-en-16-amido)phenyl)propyl (*S*)-1-(3,3-dimethyl-2-oxopentanoyl)piperidine-2-carboxylate (SP3-Acry). 4 mg. 40%. ^{1}H NMR (600 MHz, $CDCl_3$) δ 8.64 (bs, 1H), 7.60 – 7.54 (m, 2H), 7.29 (t, *J* = 7.8 Hz, 1H), 7.04 (d, *J* = 7.7 Hz, 1H), 6.79 – 6.75 (m, 1H), 6.71 – 6.65 (m, 2H), 6.36 (bs, 1H), 6.27 (d, *J* = 17.1 Hz, 1H), 6.09 (dd, *J* = 17.1, 10.3 Hz, 1H), 5.78 (dd, *J* = 8.0, 5.4 Hz, 1H), 5.61 (d, *J* = 10.3 Hz, 1H), 5.33 – 5.29 (m, 1H), 3.88 – 3.82 (m, 8H), 3.72 – 3.66 (m, 4H), 3.66 – 3.61 (m, 2H), 3.60 – 3.55 (m, 2H), 3.53 (t, *J* = 5.2 Hz, 2H), 3.48 – 3.45 (m, 2H), 3.38 – 3.33 (m, 1H), 3.20 – 3.13 (m, 1H), 2.65 (t, *J* = 5.7 Hz, 2H), 2.63 – 2.57 (m, 1H), 2.57 – 2.51 (m, 1H), 2.38 – 2.34 (m, 1H), 2.27 – 2.20 (m, 1H), 2.10 – 2.04 (m, 1H), 1.77 – 1.63 (m, 5H), 1.49 – 1.41 (m, 1H), 1.41 – 1.33 (m, 1H), 1.23 (s, 3H), 1.22 (s, 3H), 0.89 (t, *J* = 7.5 Hz, 3H). ^{13}C NMR (151 MHz, $CDCl_3$) δ 208.2, 169.9, 169.7, 169.6, 167.1, 148.9, 147.3, 140.7, 138.7, 133.5, 130.7, 129.1, 126.6, 121.9, 120.2, 119.6, 117.9, 111.8, 111.3, 76.6, 70.5, 70.3 (2C), 70.1, 69.7, 67.1, 55.9, 55.8, 51.3, 46.7, 44.2, 39.4, 38.2, 37.9, 32.5, 31.2, 26.3, 25.0, 23.4, 23.2, 21.1, 8.7. HRMS (ESI) (m/z) $[M+Na]^+$ C42H59N3O11 calc. 804.4042, found 804.4037.

Synthesis of UNC8153.

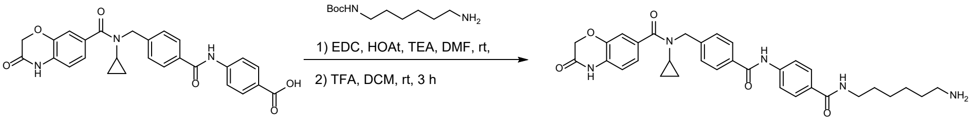

5 UNC8153

N-(4-((4-((6-aminohexyl)carbamoyl)phenyl)carbamoyl)benzyl)-N-cyclopropyl-3-oxo-3,4-dihydro-2H-benzo[b][1,4]oxazine-7-carboxamide 2,2,2-trifluoroacetate (UNC8153). Synthesized according to reported proccedure.[1] Briefly, a mixture of carboxylic acid **5** (100 mg, 0.2 mmol, 1.0 eq.), tert-butyl (6-aminohexyl)carbamate (67 mg, 0.3 mmol, 1.5 eq.), HOAt (42 mg, 0.3 mmol, 1.5 eq.), EDC (59 mg, 0.3 mmol 1.5 eq.) and triethylamine (112 µL, 0.8 mmol, 4.0 eq) in DMF (0.5 M) is stirred at room temperature until completion for 2 hours. The reaction is diluted with ethyl acetate and washed with water, sat. NH_4Cl and brine. The organic layer is dried over anhydrous Na_2SO_4, filtered and concentrated under reduced pressure. After purification by flash-chromatography using dichloromethane/methanol, the corresponding Boc-protected molecule is diluted in dichloromethane and trifluoroacetic acid is added at 0 C and the reaction stirred at rt for 3 h. Volatiles are removed under

reduced pressure and the desired compound purified by flash chromatography using dichloromethane/methanol. 60 mg. 40%. Characterization data correspond to those previously reported. ^{1}H NMR (600 MHz, MeOD) δ 7.96 (d, J = 8.3 Hz, 2H), 7.85 – 7.83 (m, 4H), 7.50 (d, J = 7.9 Hz, 2H), 7.20 (d, J = 8.1 Hz, 1H), 7.17 (d, J = 1.8 Hz, 1H), 6.96 (d, J = 8.0 Hz, 1H), 4.83 (s, 2H), 4.62 (s, 2H), 3.40 (t, J = 7.1 Hz, 2H), 2.93 (t, J = 7.6 Hz, 2H), 2.87 – 2.79 (m, 1H), 1.72 – 1.64 (m, 4H), 1.50 – 1.41 (m, 4H), 0.69 – 0.61 (m, 2H), 0.60 – 0.51 (m, 2H). MS (ESI) (m/z) $[M+H]^{+}$ C33H38N5O5 calc. 584.29, found 584.33.

^{1}H-NMR and ^{13}C-NMR spectra of SP3CHO.

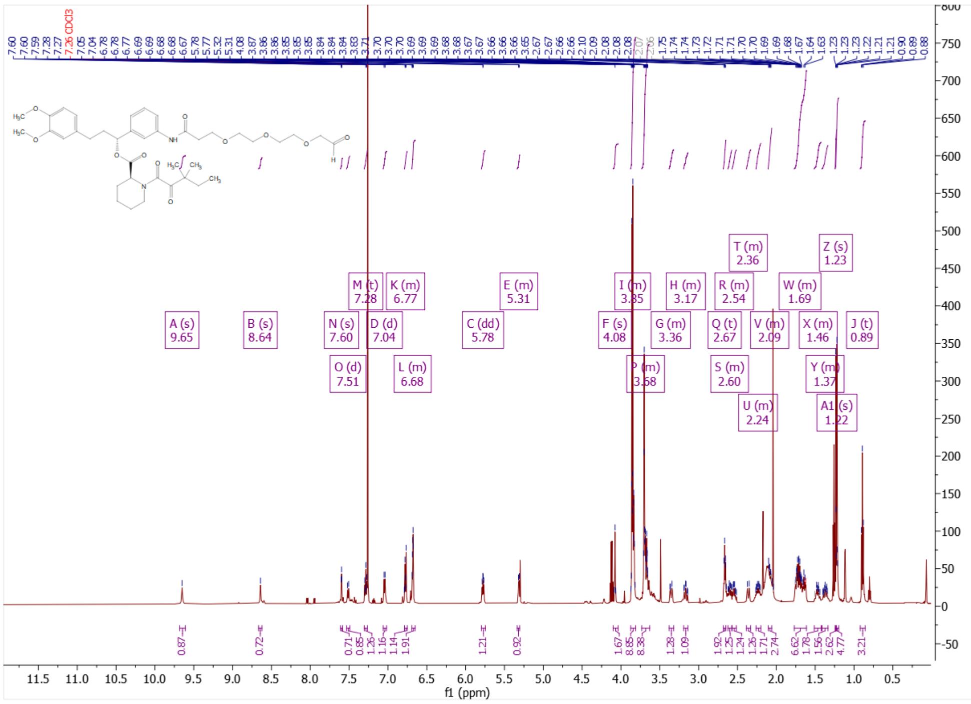

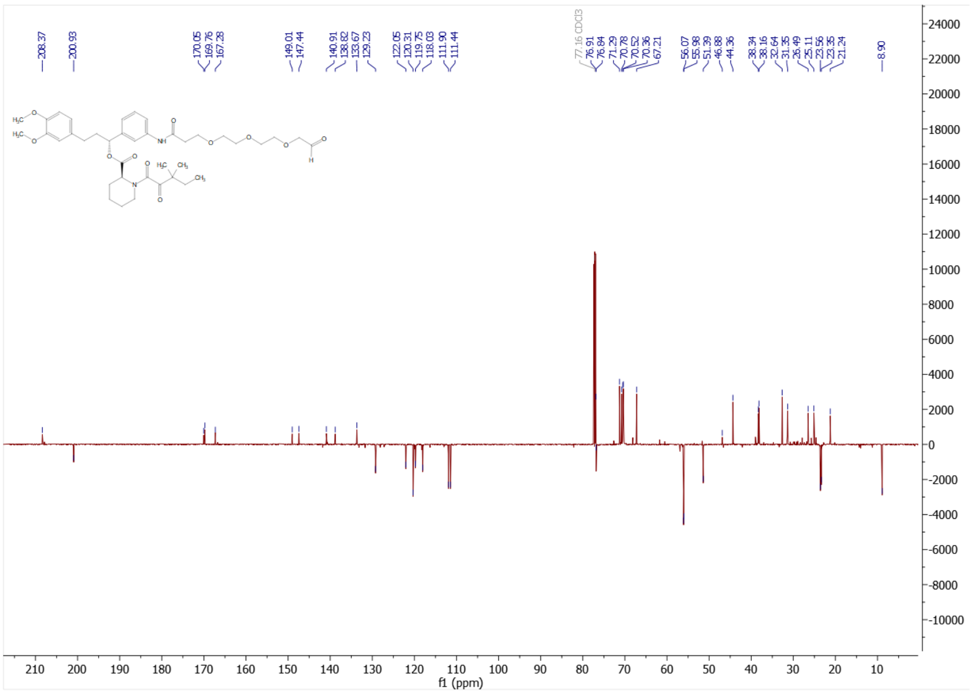

Supplementary References

1. Hanley, R. P. *et al.* Discovery of a Potent and Selective Targeted NSD2 Degrader for the Reduction of H3K36me2. *J Am Chem Soc* **145**, 8176–8188 (2023).
