# Supplementary material for: Alkylamine-tethered molecules recruit FBXO22 for targeted protein degradation
Source: Nat Commun. 2024 Jun 26;15:5409. doi: 10.1038/s41467-024-49739-3 (PMC11208438; doi:10.1038/s41467-024-49739-3)
Supplement: Supplementary file 3 — Description of Additional Supplementary Files [file 41467_2024_49739_MOESM3_ESM.pdf]

Description of Additional Supplementary Files

Supplementary Data 1: Global Proteomics statistics.

Supplementary Data 2: UPS-focused sgRNA library

Supplementary Data 3: FACS-based CRISPR/Cas9 knockout screen raw counts and statistics

Supplementary Data 4: TMT-ABPP raw peptides and statistics.
