# Supplementary material for: Alkylamine-tethered molecules recruit FBXO22 for targeted protein degradation
Source: Nat Commun. 2024 Jun 26;15:5409. doi: 10.1038/s41467-024-49739-3 (PMC11208438; doi:10.1038/s41467-024-49739-3)
Supplement: Supplementary file 8 — Reporting Summary [file 41467_2024_49739_MOESM8_ESM.pdf]

nature portfolio

Corresponding author(s): Georg E. Winter

Last updated by author(s): Jun 6, 2024

Reporting Summary

Nature Portfolio wishes to improve the reproducibility of the work that we publish. This form provides structure for consistency and transparency in reporting. For further information on Nature Portfolio policies, see our Editorial Policies and the Editorial Policy Checklist.

Statistics

For all statistical analyses, confirm that the following items are present in the figure legend, table legend, main text, or Methods section.

n/a	Confirmed	
[ ]	[x]	The exact sample size (n) for each experimental group/condition, given as a discrete number and unit of measurement
[ ]	[x]	A statement on whether measurements were taken from distinct samples or whether the same sample was measured repeatedly
[ ]	[x]	The statistical test(s) used AND whether they are one- or two-sided<br>*Only common tests should be described solely by name; describe more complex techniques in the Methods section.*
[x]	[ ]	A description of all covariates tested
[ ]	[x]	A description of any assumptions or corrections, such as tests of normality and adjustment for multiple comparisons
[ ]	[x]	A full description of the statistical parameters including central tendency (e.g. means) or other basic estimates (e.g. regression coefficient) AND variation (e.g. standard deviation) or associated estimates of uncertainty (e.g. confidence intervals)
[ ]	[x]	For null hypothesis testing, the test statistic (e.g. F, t, r) with confidence intervals, effect sizes, degrees of freedom and P value noted<br>*Give P values as exact values whenever suitable.*
[x]	[ ]	For Bayesian analysis, information on the choice of priors and Markov chain Monte Carlo settings
[x]	[ ]	For hierarchical and complex designs, identification of the appropriate level for tests and full reporting of outcomes
[x]	[ ]	Estimates of effect sizes (e.g. Cohen's d, Pearson's r), indicating how they were calculated

Our web collection on statistics for biologists contains articles on many of the points above.

Software and code

Policy information about availability of computer code

Data collection	NanoBiT assays: Multilabel Plate Reader Platform Victor X3; PerkinElmer 2030 software v4.0.<br>Western blotting/SDS gels: ChemiDoc Touch imaging system (BioRad) operated on Image Lab (v2.4.0.03).<br>Mass spectrometry - global proteomics: Orbitrap Fusion Lumos instrument coupled to a Proxeon NanoLC-1200 UHPLC. Data acquired using the FAIMSpro interface.<br>Mass spectrometry - ABPP: Orbitrap Fusion Eclipse instrument coupled to a Proxeon NanoLC-1200 UHPLC. Data acquired using the FAIMSpro interface.<br>Next generation sequencing: HiSeq 3500 platform (Illumina) (https://www.illumina.com/).<br>Flow cytometry: Data was collected on a LSRFortessa (BD Biosciences) using BD FACSDiva software (v9.0).<br>Ultra-performance liquid chromatography-mass spectrometry (UPLC-MS/MS): UHPLC system coupled to a Waters Xevo TQMS triple quadrupole mass spectrometer.<br>NanoDSF: Protein stability instrument NanoTemper Prometheus.<br>Intact-mass experiments: microTOF Bruker Daltonik instrument equipped with an Agilent 1100 HPLC system.
Data analysis	Data processing and statistical analyses: Microsoft Excel for Microsoft 365 MSO (Version 2403 Build 16.0.17425.20176), R Studio (v2023.12.1 Build 402) with R (v4.3.2), GraphPad Prism (v10.2.2).<br>Expression proteomics: Detailed analysis of expression proteomics data is described in the methods section. Acquired data were searched utilizing the open-source Comet algorithm (release_2019010) using a previously described informatics pipeline (Huttlin et al., Cell, 2010; Elias & Gygi, Nat Methods, 2007; Beausoleil et al., Nat Biotechnol, 2006; McAlister et al., Anal Chem, 2012).<br>TMT-ABPP analysis (detailed analysis described in methods section): Raw files searching with the Comet search engine (ver. 2019.01.5) and the Uniprot human proteome database; Cysteine-modified peptides filtering for site localization: AScorePro algorithm (Beausoleil et al., Nat

Biotechnol, 2006; Gassaway et al., Nat Methods, 2022).
Intact-mass experiments: Raw MS data was analyzed in CompassTM Data Analysis software from Bruker Daltonik and deconvoluted with "Maximum Entropy".
UPLC-MS/MS quantification: software TargetLynx XS V4 S2 SCN986.
Flow cytometry analysis: FlowJo (v10.8.1).
Processing of CRISPR functional genetic screening data: https://zenodo.org/records/11445611
Statistical analysis of CRISPR functional genetic screening data using MAGeCK: https://zenodo.org/records/11445588. Used packages: fastx-toolkit (v0.0.14), Bowtie2 (v2.4.5), featureCounts (v2.0.1), MAGeCK (v0.5.9).
AlphaFold: model of FBXO22 from https://alphafold.ebi.ac.uk/entry/Q8NEZ5 created with the AlphaFold Monomer v2.0 pipeline.

For manuscripts utilizing custom algorithms or software that are central to the research but not yet described in published literature, software must be made available to editors and reviewers. We strongly encourage code deposition in a community repository (e.g. GitHub). See the Nature Portfolio guidelines for submitting code & software for further information.

Data

Policy information about availability of data

All manuscripts must include a data availability statement. This statement should provide the following information, where applicable:

- Accession codes, unique identifiers, or web links for publicly available datasets
- A description of any restrictions on data availability
- For clinical datasets or third party data, please ensure that the statement adheres to our policy

The mass spectrometry data for the global proteomics generated in this study have been deposited in the ProteomeXchange Consortium via the PRIDE partner repository with the accession identifier PXD049330. The mass spectrometry data for the TMT-ABPP experiment have been deposited in the ProteomeXchange Consortium with the accession identifier PXD051803. Source data of all graphs and uncropped gels and blots are provided in the "Source Data" file. Source data for Fig. 1e, Fig. 2a, Supplementary Fig. 2a and Supplementary Fig. 4g, h are included in the Supplementary Data 1-4. The gating strategies applied for FACS analyses and cell sorting are provided in Supplementary Fig. 6.

Research involving human participants, their data, or biological material

Policy information about studies with human participants or human data. See also policy information about sex, gender (identity/presentation), and sexual orientation and race, ethnicity and racism.

Reporting on sex and gender	*Use the terms sex (biological attribute) and gender (shaped by social and cultural circumstances) carefully in order to avoid confusing both terms. Indicate if findings apply to only one sex or gender; describe whether sex and gender were considered in study design; whether sex and/or gender was determined based on self-reporting or assigned and methods used.*<br>*Provide in the source data disaggregated sex and gender data, where this information has been collected, and if consent has been obtained for sharing of individual-level data; provide overall numbers in this Reporting Summary. Please state if this information has not been collected.*<br>*Report sex- and gender-based analyses where performed, justify reasons for lack of sex- and gender-based analysis.*
Reporting on race, ethnicity, or other socially relevant groupings	*Please specify the socially constructed or socially relevant categorization variable(s) used in your manuscript and explain why they were used. Please note that such variables should not be used as proxies for other socially constructed/relevant variables (for example, race or ethnicity should not be used as a proxy for socioeconomic status).*<br>*Provide clear definitions of the relevant terms used, how they were provided (by the participants/respondents, the researchers, or third parties), and the method(s) used to classify people into the different categories (e.g. self-report, census or administrative data, social media data, etc.)*<br>*Please provide details about how you controlled for confounding variables in your analyses.*
Population characteristics	*Describe the covariate-relevant population characteristics of the human research participants (e.g. age, genotypic information, past and current diagnosis and treatment categories). If you filled out the behavioural & social sciences study design questions and have nothing to add here, write "See above."*
Recruitment	*Describe how participants were recruited. Outline any potential self-selection bias or other biases that may be present and how these are likely to impact results.*
Ethics oversight	*Identify the organization(s) that approved the study protocol.*

Note that full information on the approval of the study protocol must also be provided in the manuscript.

Field-specific reporting

Please select the one below that is the best fit for your research. If you are not sure, read the appropriate sections before making your selection.

☒ Life sciences ☐ Behavioural & social sciences ☐ Ecological, evolutionary & environmental sciences

For a reference copy of the document with all sections, see nature.com/documents/nr-reporting-summary-flat.pdf

Life sciences study design

All studies must disclose on these points even when the disclosure is negative.

Sample size	All experiments were performed on cultured human cell lines or in vitro. Sample sizes were not pre-determined with statistical analyses, but were based on prior experience in the field and our previous studies (Hsia et al, Nature, 2024; Mayor-Ruiz et al, Mol Cell, 2019; Brand et al, Cell Chem Biol, 2019; Mayor-Ruiz et al, Nat Chem Biol, 2020, Kuljanin et al., Nat Biotechnol, 2021; Yang et al., Cell Chem Biol, 2024, Adolf et al, Nat Struct Mol Biol. 2024).
Data exclusions	No data was excluded.
Replication	Attempts for replication were successful. Unless stated otherwise, experiments were repeated at least twice for reproducibility. The number of technical or biological replicates are specified in the respective figure legends.
Randomization	Samples undergoing compound treatments were randomized. Prior to treatment, they were numerically labeled and randomly assigned. Post-treatment, the samples were processed randomly in a single batch. All experiments incorporated both positive and negative controls, and orthogonal methods were employed to verify the results.
Blinding	Not relevant to this study. No subjective measurements were performed.

Reporting for specific materials, systems and methods

We require information from authors about some types of materials, experimental systems and methods used in many studies. Here, indicate whether each material, system or method listed is relevant to your study. If you are not sure if a list item applies to your research, read the appropriate section before selecting a response.

Materials & experimental systems

n/a	Involved in the study	
☐	☒	Antibodies
☐	☒	Eukaryotic cell lines
☒	☐	Palaeontology and archaeology
☒	☐	Animals and other organisms
☒	☐	Clinical data
☒	☐	Dual use research of concern
☒	☐	Plants

Methods

n/a	Involved in the study	
☒	☐	ChIP-seq
☐	☒	Flow cytometry
☒	☐	MRI-based neuroimaging

Antibodies

Antibodies used	Western blot:<br>Primary antibodies: anti-αTubulin DM1A (T9026, Sigma-Aldrich, 1:5000), anti-NSD2 29D1 (Ab-75357, Abcam, 1:1000), anti-FKBP12 H-5 (sc-133067, 1:1000), anti-FBXO22 FF-7 (sc-100736, 1:400), anti-XIAP E-2 (sc-55551, 1:200) and anti-GADPH 0411 (sc-47724, 1:5000) all purchased from SantaCruz Biotechnology, anti-cMYC D84C12 (#5605, 1:1000), anti-HA-Tag C29F4 (#3724, 1:1000), anti-BRD4 E2A7X (#13440, 1:2000), anti-CRBN D8H3S (#71810, 1:1000) and anti-V5 D3H8Q (#13202, 1:1000) from Cell Signaling Technology, anti-Flag M2 (F1804, Sigma-Aldrich, 1:1000) and anti-BRD3 (#A302-368A, Bethyl Laboratories, 1:1000). Secondary antibodies used: anti-rabbit IgG, HRP-linked (#7074, 1:10000) and anti-mouse IgG, HRP-linked (#7076, 1:10000) both from Cell Signaling Technology.<br>FACS sorting: anti-CD90.1/Thy-1.1-APC (202526, Biolegend, 1:400)
Validation	Validations and references for all antibodies can be found in the vendor sites using the above specified catalogue numbers.<br>Additional validations in this manuscript:<br>For NSD2 and XIAP validated based on expected reduction of protein levels upon treatments with degraders (Fig. 4f), as shown by Hanley et al., J. Am. Chem. Soc., 2023 and den Besten et al., J. Am. Chem. Soc., 2021, respectively.<br>For anti-FKBP12: expected loss upon SP3N treatment (Fig. 1d) & expected loss by known degrader PROTAC dFKBP1 (Winter et al., Science, 2015).<br>For anti-FBXO22: loss of protein in FBXO22 knock-out cells (Supplementary Fig. 2b).<br>For anti-BRD3 and anti-BRD4: expected loss of protein with treatment with dBEt6 degrader PROTAC as shown by Winter et al., Mol. Cell, 2017.<br>For HA-tag, Flag-tag and V5-tag: in HA-tagged-FBXO22 and Flag-tagged-FKBP12 expressing cell lines (Fig. 2d), in cells transfected with HA-Tagged-CRBN and V5-tagged BRD4s (Supplementary Fig. 5c).

Eukaryotic cell lines

Policy information about cell lines and Sex and Gender in Research

Cell line source(s)	KBM7 inducible Cas9 (iCas9) cells were a gift from Johannes Zuber / IMP - Research Institute of Molecular Pathology. 293T

were from ATCC (CRL-3216) and Lenti-X 293T from Clontech (632180).
All other cell lines used in this study were generated using any of the parental cell lines above.

Authentication

Cell lines were authenticated by vendors and routinely authenticated via cell morphology.

Mycoplasma contamination

All cell lines were routinely tested and confirmed negative for mycoplasma contamination.

Commonly misidentified lines
(See ICLAC register)

No commonly misidentified cell lines were used.

Plants

Seed stocks

Report on the source of all seed stocks or other plant material used. If applicable, state the seed stock centre and catalogue number. If plant specimens were collected from the field, describe the collection location, date and sampling procedures.

Novel plant genotypes

Describe the methods by which all novel plant genotypes were produced. This includes those generated by transgenic approaches, gene editing, chemical/radiation-based mutagenesis and hybridization. For transgenic lines, describe the transformation method, the number of independent lines analyzed and the generation upon which experiments were performed. For gene-edited lines, describe the editor used, the endogenous sequence targeted for editing, the targeting guide RNA sequence (if applicable) and how the editor was applied.

Authentication

Describe any authentication procedures for each seed stock used or novel genotype generated. Describe any experiments used to assess the effect of a mutation and, where applicable, how potential secondary effects (e.g. second site T-DNA insertions, mosiacism, off-target gene editing) were examined.

Flow Cytometry

Plots

Confirm that:

- [x] The axis labels state the marker and fluorochrome used (e.g. CD4-FITC).
- [x] The axis scales are clearly visible. Include numbers along axes only for bottom left plot of group (a 'group' is an analysis of identical markers).
- [x] All plots are contour plots with outliers or pseudocolor plots.
- [x] A numerical value for number of cells or percentage (with statistics) is provided.

Methodology

Sample preparation

For sorting of CRISPR/Cas9 screen: The screen cells were expressing inducible Cas9-GFP, the FKBP12-BFP-P2A-mCherry fluorescent reporter and were transduced with the sgRNA library cloned into the pLentiV1-PBS69-U6-sgRNA-IT-EF1as-Thy1.1-P2A-Neo vector. 3 days post doxycycline induction and 16h post-compound treatments, the cells were incubated with anti-CD90.1/Thy-1.1-APC (202526, Biolegend, 1:400), Zombie NIR™ Fixable Viability Dye (BioLegend, 1:1000) and Human TruStain FcX™ Fc Receptor Blocking Solution (1:400, BioLegend), for 10 min at 4 oC, fixed with BD Fixation buffer 4% (Thermo Scientific™ Pierce™) for 45 min at 4 oC, protected from light and stored in PBS + 5% FCS + 1 mM EDTA overnight at 4 oC. The next day, the cells were sorted on a BD FACSAria™ Fusion (BD Biosciences) using a 100 µm nozzle.

For all flow cytometry-based experiments with the stability reporters: Suspension cells expressing the FKBP12-BFP-P2A-mCherry reporter (or the BRD4-BFP-P2A-mCherry reporter) were collected after the respective treatments and directly measured for their BFP/mCherry levels. Adherent cells were detached using trypsin and resuspended in cell culture media before measuring. Flow cytometry measurements were performed with BD LSRFortessa™ Cell Analyzer (BD Biosciences).

To engineer a clonal FBXO22 knock out cell line, HEK293T FKBP12-BFP-P2A-mCherry cells were transduced with plasmids expressing the sgRNA 'GATCCAGGTTACGCTCCGAT' targeting FBXO22. Post G418 selection, the cells were transfected with pSpCas9(BB)-2A-Puro (PX459) v2.0 (Addgene #62988) plasmid using PEI. 48 h post transfection, cells were trypsinized, resuspended in media and the BFP+mCherry+ single clones were seeded in 96 well plates using CytoFLEX LX sorter (Beckman Coulter) and grown for 2 weeks at 37 oC and 5% CO2 humidified incubator.

Instrument

Flow cytometric analyses: BD LSRFortessa (4 laser, 16 detector configuration; BD Bioscience).
Sorting: BD FACSAria Fusion (5 lasers, 16 detectors; BD Bioscience).
Single cell sorting for single FBXO22 knock out clones: CytoFLEX SRT (4 lasers, 15 detectors; Beckman Coulter).

Software

BD FACSDiva software (v9.0), Beckman Coulter CytExpert SRT (v 1.1.0.10007), FlowJo (v10.8.1)

Cell population abundance

Screen sorting: The fractions of FKBP12-HIGH (5-8%), FKBP12-MID (30-35%) and FKBP12-LOW (5-8%) were sorted based on the FKBP12-BFP-mCherry expression levels. To achieve more than 1200x library representation, at least 500.000 cells were collected in the FKBP12-HIGH and LOW fractions, and more than 3 million cells in the FKBP12-MID population. The purity was determined as >95% with post-sorting quality control: a small aliquot of each fraction was measured at BD LSRFortessa™ Cell Analyzer (BD Biosciences) and the overlap of the different fractions was calculated.

Gating strategy

For the CRISPR screen sorting (Supplementary Fig. 6a): forward scatter area and side scatter area were used to separate cell events from debri and the aggregates were excluded using the forward scatter height and side scatter area as well as side

scatter height and side scatter width. The live cells (ZombieNIR-BV786-) were gated for iCas9-GFP+CD90-APC+. In this population, the fractions FKBP12-HIGH (5-8%), FKBP12-MID (~30%) and FKBP12-LOW (5-8%) were sorted based on the BFP and mCherry levels.
For all the rest flow cytometry based measurements for BFP-mCherry levels (Supplementary Fig. 6b), the debri and aggregates were excluded as above based on the forward scatter and side scatter and the BFP/mCherry levels were determined.

☒ Tick this box to confirm that a figure exemplifying the gating strategy is provided in the Supplementary Information.
